# Supplementary material for: Interferon-driven brain phenotype in a mouse model of RNaseT2 deficient leukoencephalopathy
Source: Nat Commun. 2021 Nov 11;12:6530. doi: 10.1038/s41467-021-26880-x (PMC8586222; doi:10.1038/s41467-021-26880-x)
Supplement: Supplementary file 1 — Supplementary Information [file 41467_2021_26880_MOESM1_ESM.pdf]

Supplementary Information

Interferon-driven brain phenotype in a mouse model of RNaseT2 deficient leukoencephalopathy

Kettwig and Ternka et al.

Contents:

Supplementary Figures 1-13

Supplementary Tables 1-2

Uncropped image data

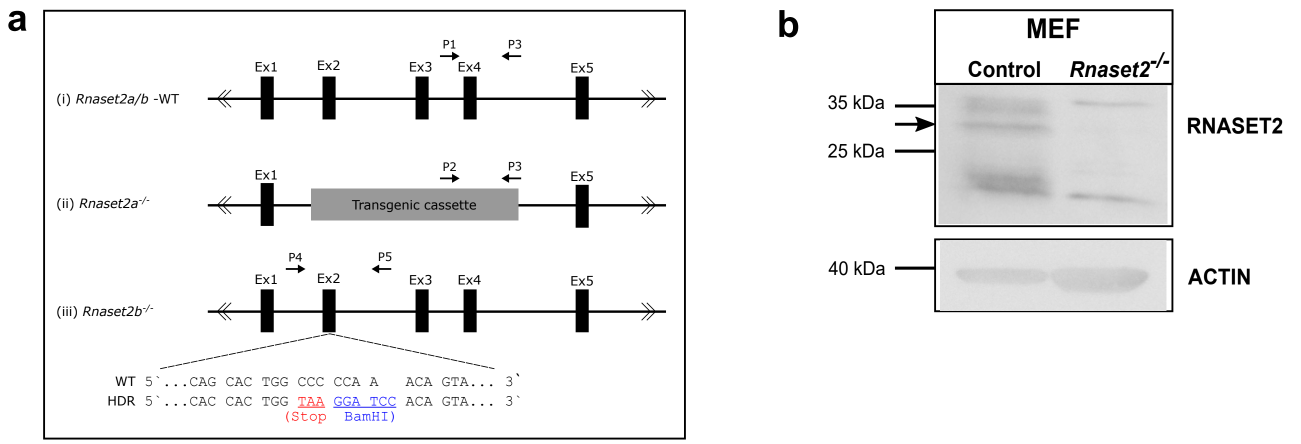

Supplementary Fig. 1 | Generation and validation of *Rnaset2*$^{-/-}$ mice. a, (I) Depiction of the relevant genomic region (Exon 1 to Exon 5, black boxes) of wild-type *Rnaset2a* and *Rnaset2b* allele (*Rnaset2a/b*-WT) for RNASET2a/b double knockout generation strategy. (II) Genomic integration of a transgenic cassette in the *Rnaset2a* coding region results in the *Rnaset2a* knockout mouse (RNT2A-KO). (III) The *Rnaset2b* allele in the RNT2A-KO mouse was genetically modified with CRISPR/Cas9. By adding a template DNA, the endogenous homology directed repair system (HDR) introduced a TAA stop codon and a *BamHI* restriction site in Exon 2 of the *Rnaset2b* gene. Arrows indicate primer pairs used for genotyping PCR and Sanger sequencing. **b**, Western blot confirming loss of RNaseT2 protein in *Rnaset2*$^{-/-}$ Mouse embryonic fibroblasts (MEF) compared to control MEFs in three independent experiments. 20 µg of total MEF protein lysate was loaded per lane and RNaseT2 specific band was revealed by using a polyclonal primary antibody (black arrow, approximately 31 kDa). Source data are provided as a Source Data file.

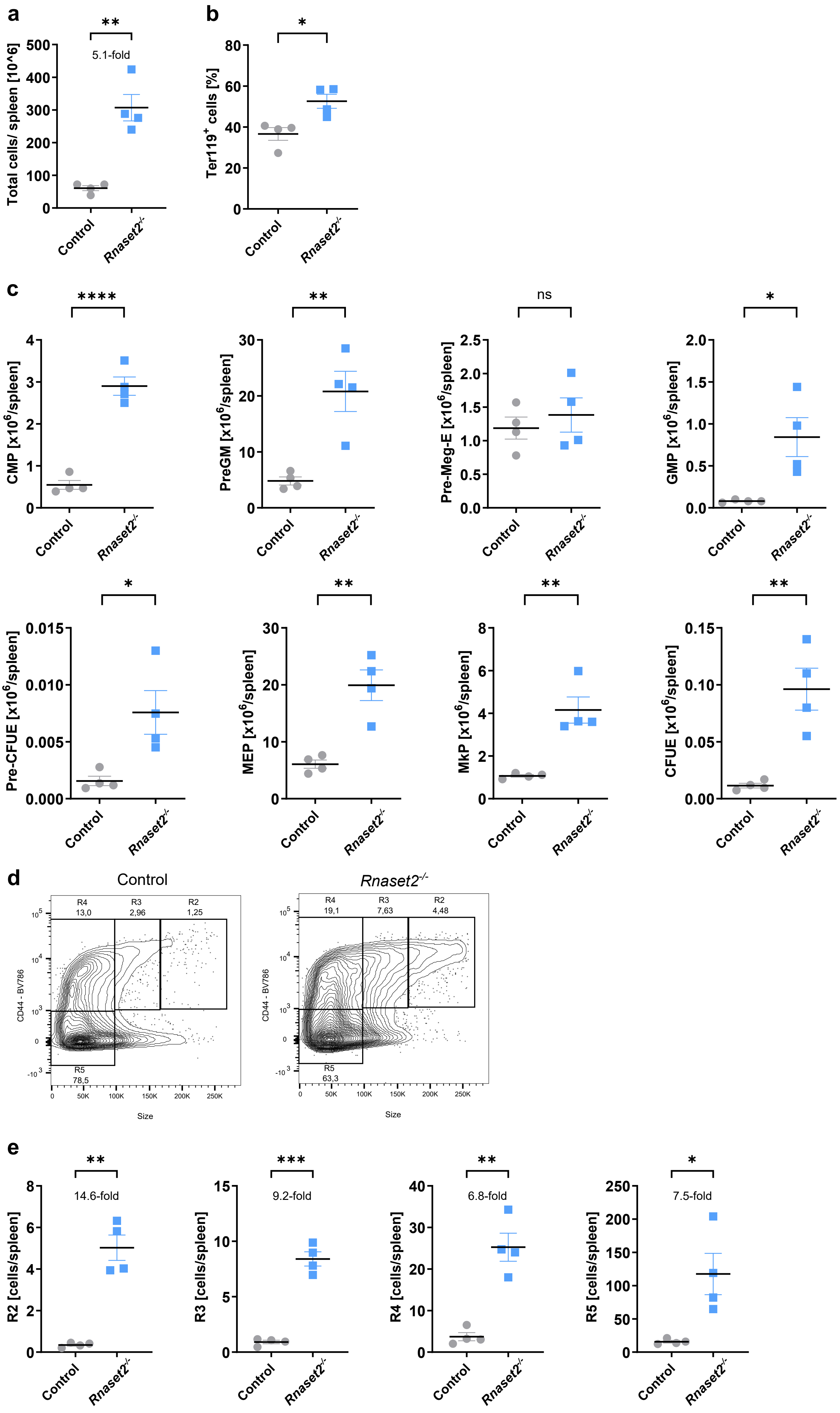

a
**
5.1-fold
Total cells/ spleen [10^6]
0
100
200
300
400
500
Control
Rnaset2-/-
b
*
Ter119+ cells [%]
0
20
40
60
80
Control
Rnaset2-/-
c
****
CMP [x10^6/spleen]
**
PreGM [x10^6/spleen]
ns
Pre-Meg-E [x10^6/spleen]
*
GMP [x10^6/spleen]
*
Pre-CFUE [x10^6/spleen]
**
MEP [x10^6/spleen]
**
MkP [x10^6/spleen]
**
CFUE [x10^6/spleen]
Control
Rnaset2-/-
d
Control
Rnaset2-/-
R4 13,0
R3 2,96
R2 1,25
R5 78,5
R4 19,1
R3 7,63
R2 4,48
R5 63,3
CD44 - BV786
Size
0
50K
100K
150K
200K
250K
e
**
14.6-fold
R2 [cells/spleen]
***
9.2-fold
R3 [cells/spleen]
**
6.8-fold
R4 [cells/spleen]
*
7.5-fold
R5 [cells/spleen]
Control
Rnaset2-/-

Supplementary Fig. 2 | Flow cytometric analyses of hematopoietic precursor cells in spleen. a, Total number of cells per spleen was 5.1-fold increased, consistent with spleen weight (*p* value of two-tailed Welsh's t-test 0.0075). **b**, Higher level of $Ter119^+$ cells in spleen indicates increased erythroid lineage (two-tailed Student's t-test *p*=0.0137). **c,** All myeloid progenitor cells from common myeloid progenitor (CMP, *p*<0.0001) over pre-granulocyte–macrophage progenitor (PreGM, *p*=0.0048) to granulocyte–macrophage progenitor (GMP, *p*=0.0169), pre-colony-forming unit erythroid (Pre-CFUE, *p*=0.0219), megakaryocyte–erythroid progenitor (MEP, *p*=0.0025) and megakaryocyte progenitor (MkP, *p*=0.0024) as well as CFUE (*p*=0.0038) were significant elevated in *Rnaset2*$^{-/-}$ with an exception to pre-megakaryocyte-erythroid (Pre-Meg-E, *p*=0.5431). *P* values of two-tailed Student's t-test are represented as *$p<0.05$, **$p<0.01$, ***$p<0.001$, ****$p<0.0001$. Data depicted as the mean ± SEM **d,** Representative FACS plots of $Ter119^+$ and $B220^-$ cells. Quantitation of erythroid fractions determined by size and CD44 expression showed a significant increase in erythroid progenitors (R2) through proerythroblasts, basophilic erythroblasts (R3), polychromatic erythroblasts, orthochromatic erythroblasts (R4) to reticulocytes (R5) as evidence of increased extramedullary erythropoiesis in the spleen. **e,** Statistical evaluation of single erythroid fractions (R2-R5) revealed a disproportionate increase in erythroid lineage compared to total cells. N=4 independent mice for all analyses. *P* values of two-tailed Welsh's t-test for R2-5 are 0.0044, 0.0009, 0.0054 and 0.0459, respectively. *P* values are represented as *$p<0.05$, **$p<0.01$, ***$p<0.001$, ****$p<0.0001$ and ns $p \geq 0.05$. Data depicted as the mean ± SEM. Source data are provided as a Source Data file.

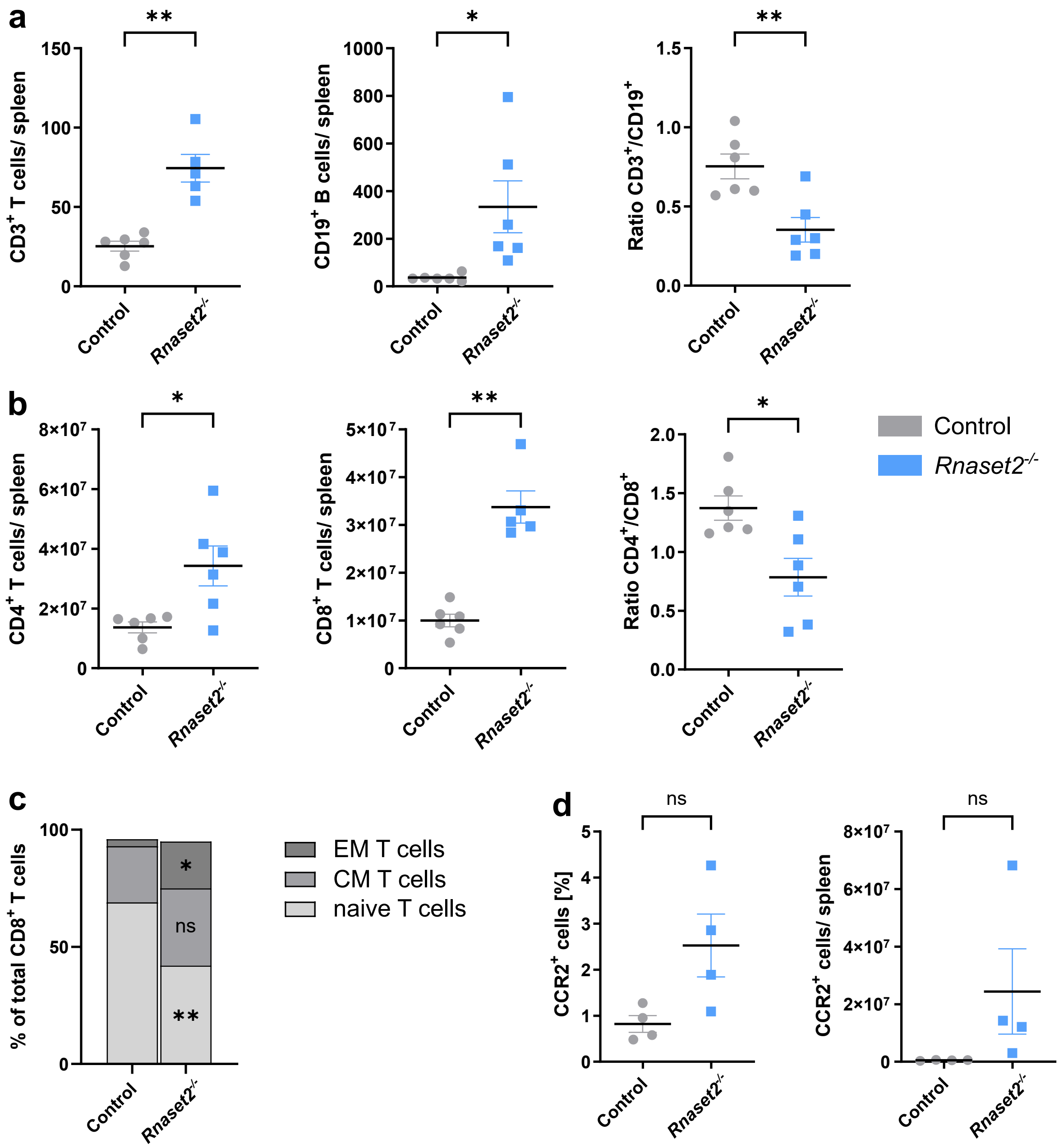

Supplementary Fig. 3 | Flow cytometric analyses of spleen lymphocytes. a, Total number of T cells (CD3⁺) (n=6 control and n=5 *Rnaset2*$^{-/-}$animals, *p*=0.0032) and B cells (CD19⁺) (n=6 *Rnaset2*$^{-/-}$ and control animals, respectively, *p*=0.0414) were increased. The ratio indicated that B cells are more increased in *Rnaset2*$^{-/-}$ mice than T cells (*p*=0.0045). **b,** Total numbers of CD4⁺ (CD45⁺ CD3⁺ CD4⁺) (n=6 *Rnaset2*$^{-/-}$and control animals, respectively, *p*=0.0264) and CD8⁺ (CD45⁺ CD3⁺ CD8⁺) (n=6 control and n=5 *Rnaset2*$^{-/-}$animals, *p*=0.0011) T cells per spleen were both significantly increased and the CD4/CD8 ratio was significantly reduced (*p*=0.0139). **c,** Proportion of CD8⁺ T cells harbouring an effector memory phenotype (CD45⁺ CD3⁺ CD8⁺ CD44^{hi} CD62L^{-}) was significantly increased (*p*=0.0444) while the proportion of naïve CD8⁺ T cells (CD45⁺ CD3⁺ CD8⁺ CD44^{low} CD62L⁺) was significantly decreased (*p*=0.0085). The proportion of central memory CD8⁺ T cells (CD45⁺ CD3⁺ CD8⁺ CD44^{hi} CD62L⁺) remain unaltered (*p*=0.1700). **d,** Depicted is the increased number of Ly6C^{hi} CCR2⁺ monocytes (CD45⁺ CD11b⁺ Ly6C^{hi} CCR2⁺) in percentage (*p*=0.0842) and absolute (*p*=0.2049) (n=4 *Rnaset2*$^{-/-}$ and control animals, respectively). *P* values of two-tailed Welsh's t-test are presented as *p<0.05, **p<0.01, ***p<0.001, ****p<0.0001 and ns p≥0.05. Data depicted as the mean ± SEM. Source data are provided as a Source Data file.

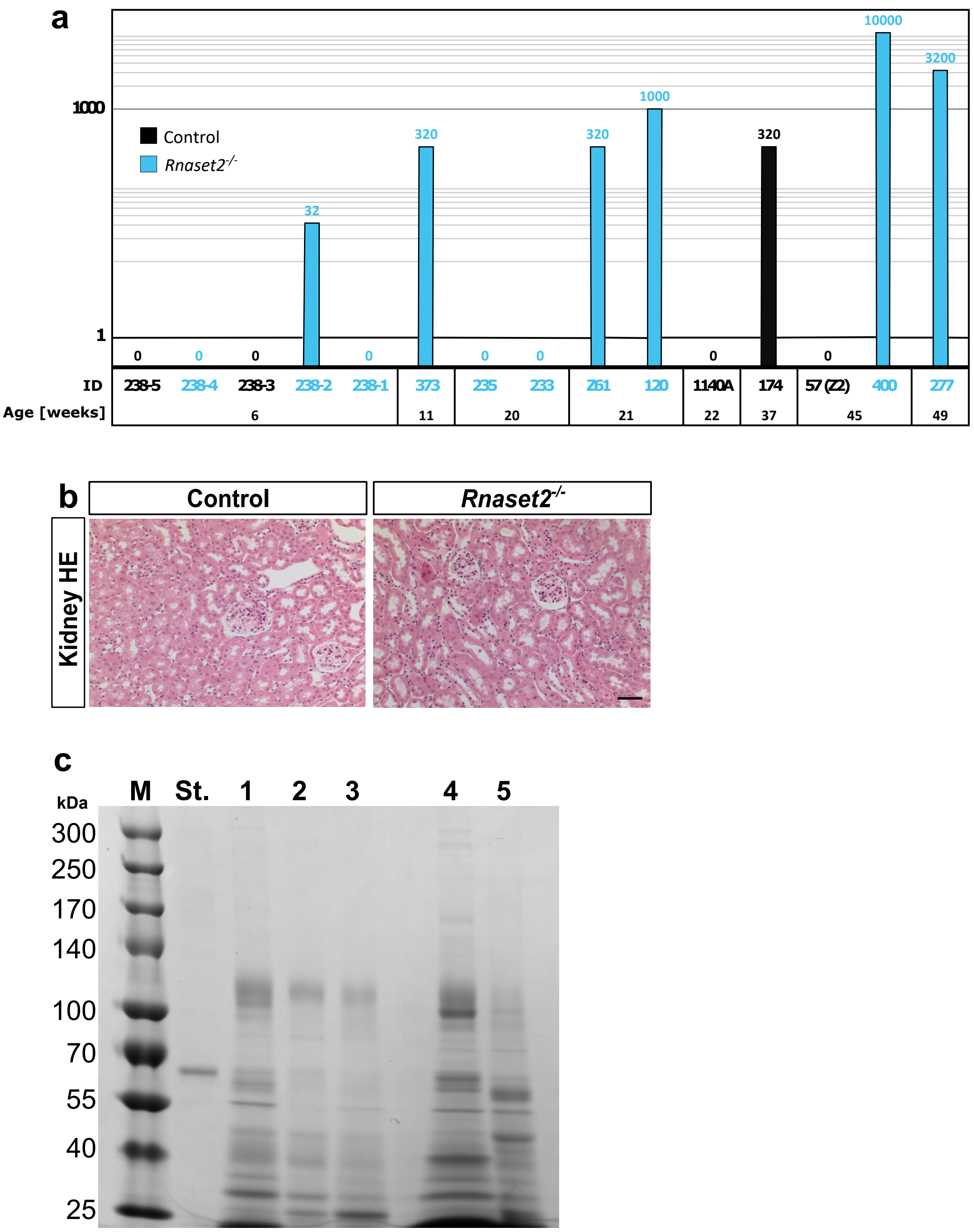

Supplementary Fig. 4 | Evaluation of autoimmunity in *Rnaset2$^{-/-}$* mice. a, Anti-nuclear antibody (ANA) titres from *Rnaset2$^{-/-}$* and control mouse sera sorted by age (weeks). Negative ANA indirect immunofluorescence test (IIFT) results are designated with ANA titres of zero (0). Positive ANA IIFT results are depicted as ratios with 1:32 (32), 1:320 (320), 1:1000 (1000), 1:3200 (3200) and 1:10000 (10000). Only *Rnaset2$^{-/-}$* sera show high titres of autoantibodies above a ratio of 1:320 till 1:10.000, indicating autoimmune phenomena. **b,** HE staining of *Rnaset2$^{-/-}$* and control mice kidney sections with *Rnaset2$^{-/-}$* mouse kidney showing no abnormalities compared to control mice. Representative images of n=7 *Rnaset2$^{-/-}$* and control mice. Scale bar 50 µm. **c,** SDS PAGE analysis of urine samples from *Rnaset2$^{-/-}$* and control mice. *Rnaset2$^{-/-}$* mice do not show elevated protein in the urine when compared to control mice, n=3 for *Rnaset2$^{-/-}$* and control mice in one experiment. From left to right: Lane 1: molecular markers (M) from 25-300 kDa, lane 2: 300 ng BSA as control (St.), lane 3-7: Urine of female control mouse (1), urine of female *Rnaset2$^{-/-}$* mouse (2,3), urine of male control mouse (4) and urine of male *Rnaset2$^{-/-}$* mouse (5). Source data are provided as a Source Data file.

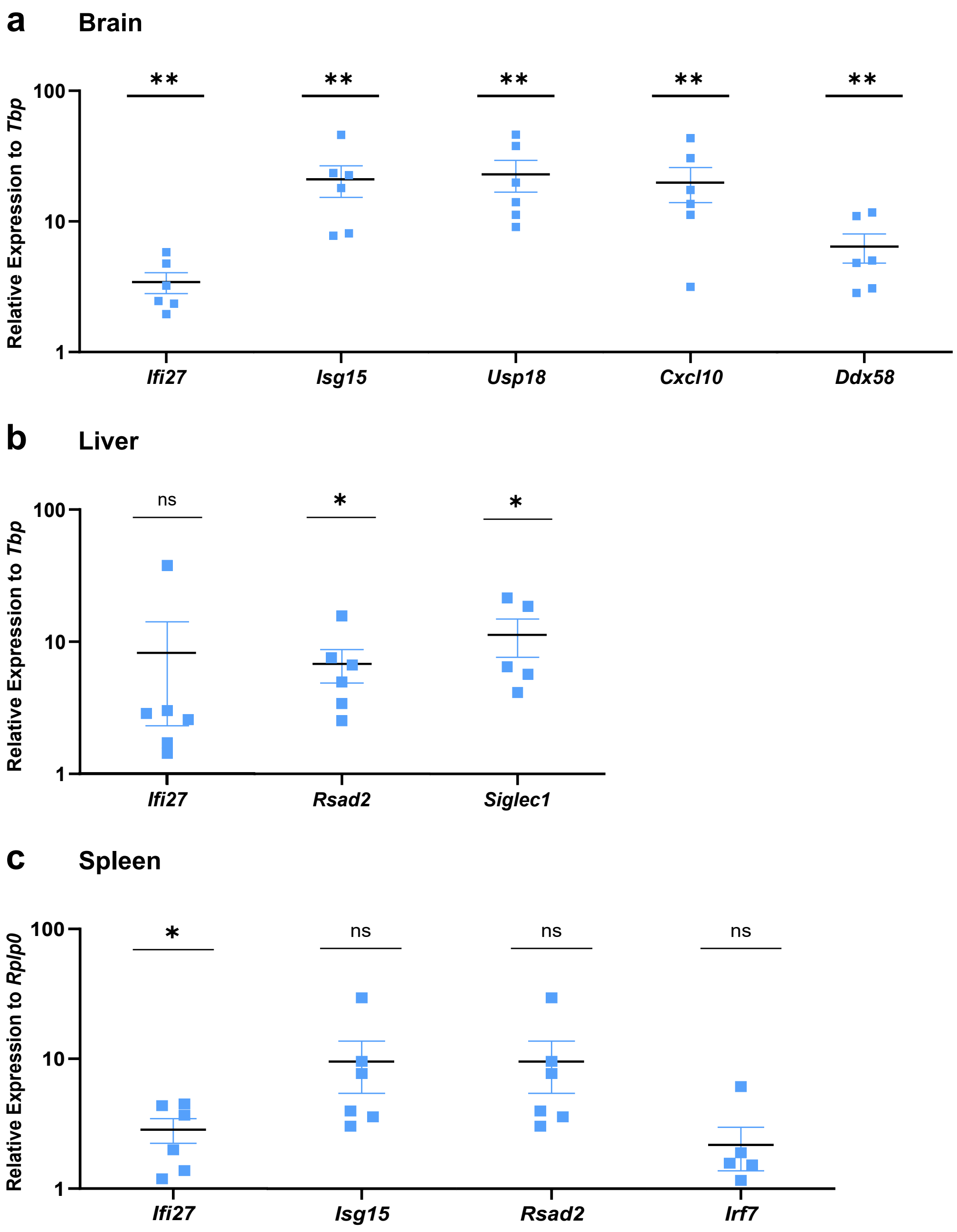

Supplementary Fig. 5 | Interferon-stimulated gene response. Quantitative PCR analyses of interferon-stimulated genes (ISGs) of various organs. The relative expression of control was normalized to 1 and to transcripts of different housekeeping genes referring to the organ. N=6 mice for *Rnaset2*$^{-/-}$ and control. *P* values of two-tailed Student's t-test are represented as **p*<0.05, ***p*<0.01, ****p*<0.001, *****p*<0.0001 and ns *p*≥0.05. Data depicted as the mean ± SEM **a,** ISGs *Ifi27* (*p*=0.0031) *Isg15* (*p*=0.0057), *Usp18* (*p*=0.0056), *Cxcl10* (*p*=0.0100) and *Ddx58* (*p*=0.0072) are substantially upregulated in the brain of *Rnaset2*$^{-/-}$. Transcripts of TATA -box-binding-protein (*Tbp*) gene was used as housekeeping gene. **b,** Liver samples showed a significant upregulation of *Rsad2* (*p*=0.0134) and *Siglec2* (*p*=0.0221) expression, relative to the control. *Tbp* was used as internal standard. *P* value for *Ifi27* is 0.2496. **c,** Upregulation of *Ifi27* (*p*=0.0131), *Isg15* (*p*=0.0642), *Rsad2* (*p*=0.0642) and *Irf7* (*p*=0.1731) were seen in the spleen. The relative expression was normalized to the transcript of the ribosomal protein *Rplp0*. Source data are provided as a Source Data file.

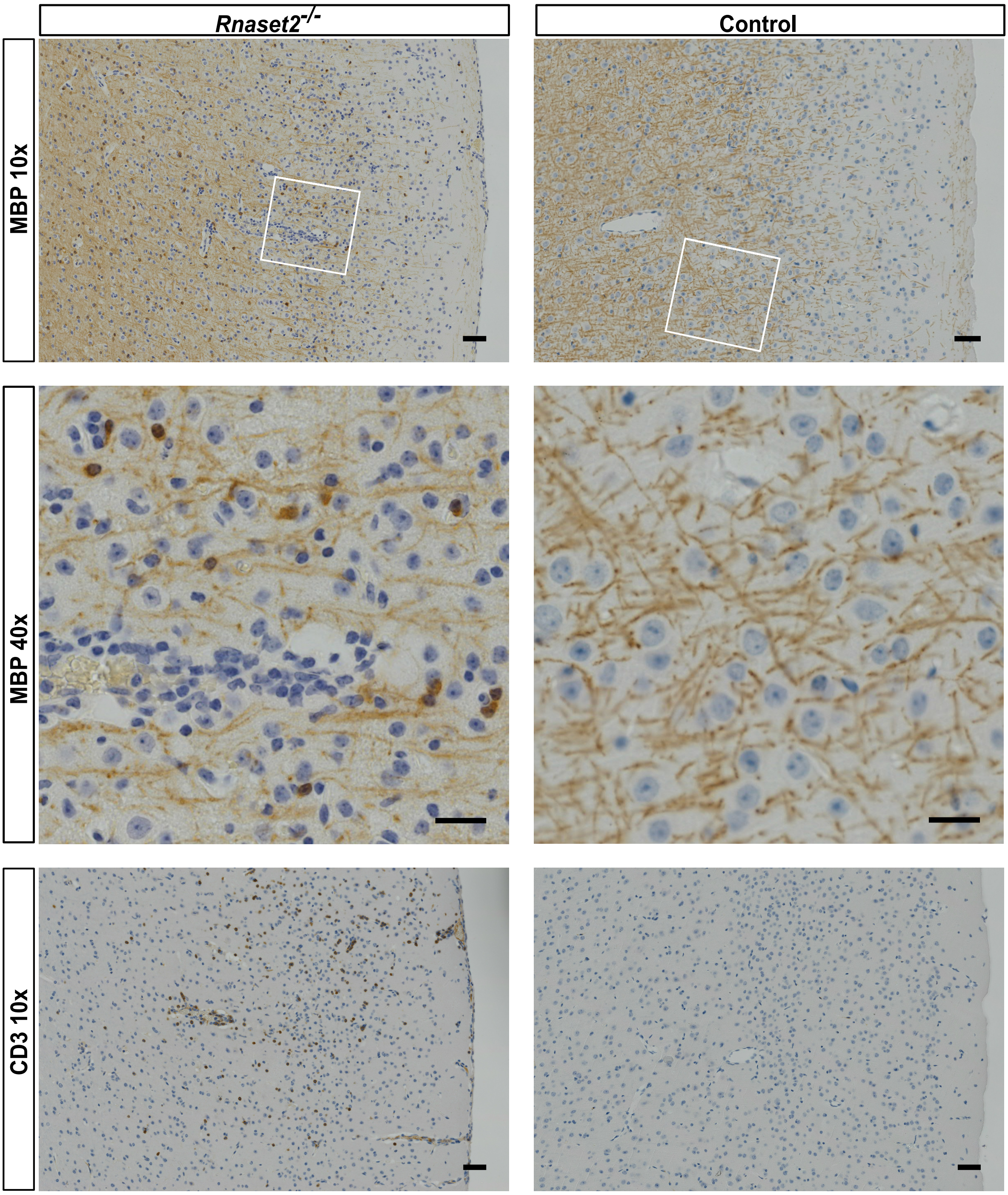

Supplementary Fig. 6 | No overt demyelination in *Rnaset2$^{-/-}$* mice compared to littermate controls demonstrated by myelin basic protein (MBP) immunohistochemistry of representative control (n=6, right column) and *Rnaset2$^{-/-}$* (n=6, left column) animals even in cortical regions with perivascular and diffuse T-cell infiltration (stratified by CD3, last row). Serial sections for CD3 and MBP are shown. CD3 and MBP are labeled in brown (DAB). 10x magnification with scale bar 50 µm first and last row, 40x magnification with scale bar 20 µm second row.

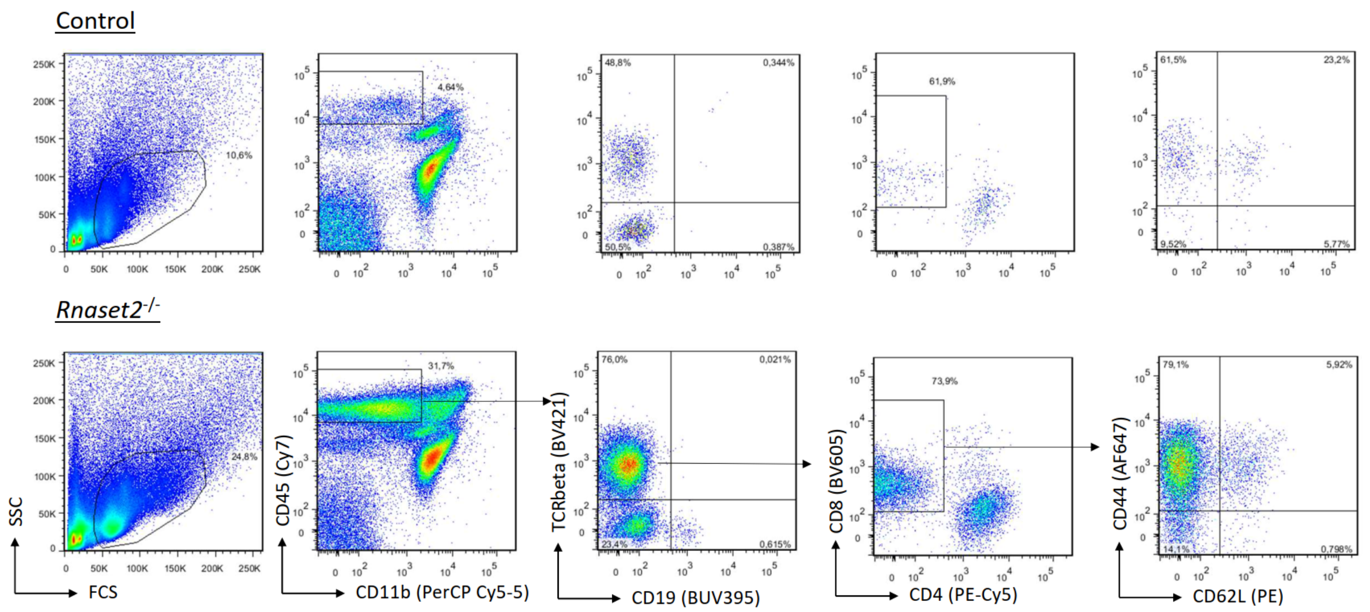

Supplementary Fig. 7 | Gating strategy for CD8⁺ T cells subtypes. Exemplary data from FACS analyses for *Rnaset2*$^{-/-}$ and control mouse brain. Naïve CD8 T cells were defined as $CD45^+$ $TCR\beta^+$ $CD8^+$ $CD44^{low}$ $CD62L^+$, effector memory CD8 T cells as $CD45^+$ $TCR\beta^+$ $CD8^+$ $CD44^{hi}$ $CD62L^-$ and central memory CD8 T cells as $CD45^+$ $TCR\beta^+$ $CD8^+$ $CD44^{hi}$ $CD62L^+$.

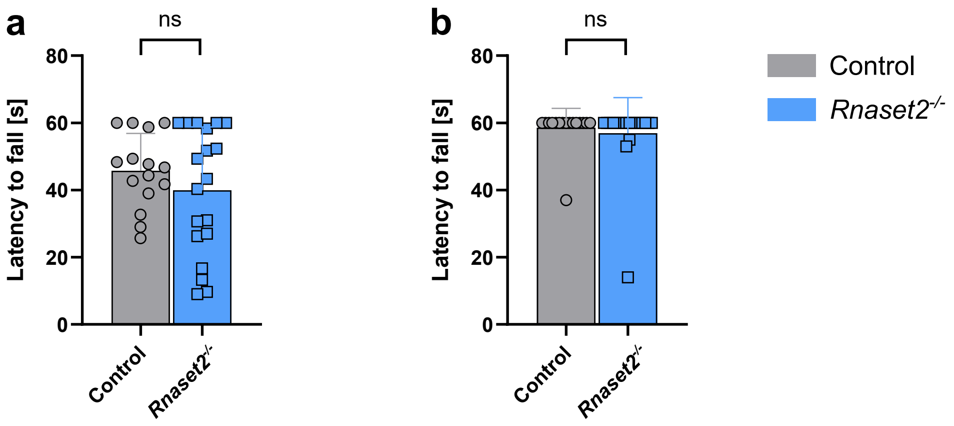

Supplementary Fig. 8 | Overall motor performance is not different in *Rnaset2*$^{-/-}$ and control mice. a, Testing of skillfulness and balance in the balance beam task revealed no differences between genotypes. N=14 control and *Rnaset2*$^{-/-}$ mice, respectively. **b,** Inverted grid hanging showed the same abilities in both genotypes. N=14 control and *Rnaset2*$^{-/-}$ mice, respectively. *P* values of two-tailed Student's t-test are depicted as 0.3011 and 0.5888 for balance beam task and inverted grid hanging. Data depicted as the mean ± SEM and *p* values are represented as **p*<0.05, ***p*<0.01, ****p*<0.001, *****p*<0.0001 and ns *p*≥0.05. Source data are provided as a Source Data file.

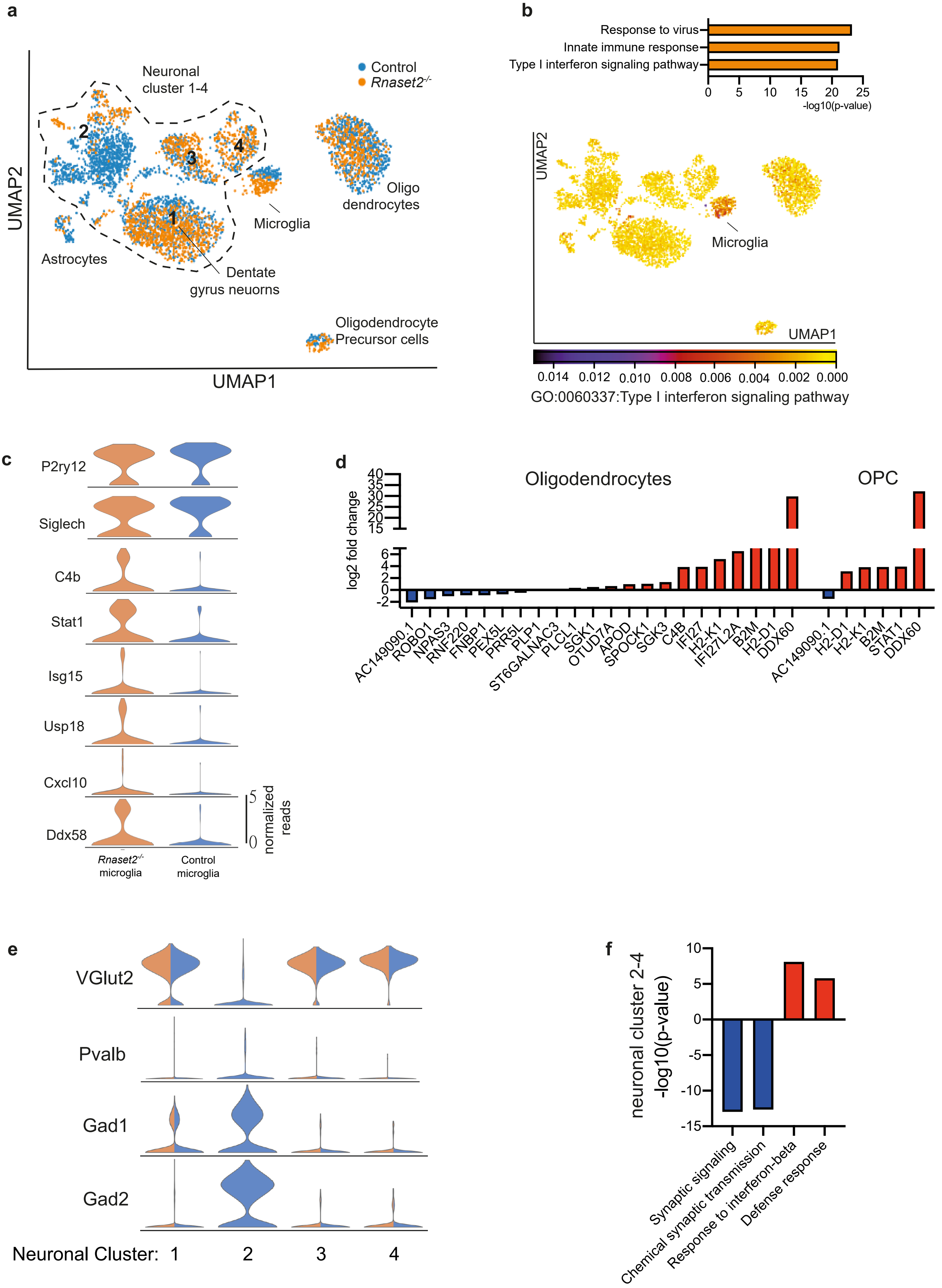
a
Neuronal cluster 1-4
Control
Rnaset2-/-
2
3
4
1
Oligo dendrocytes
Microglia
Astrocytes
Dentate gyrus neuorns
Oligodendrocyte Precursor cells
UMAP2
UMAP1
b
Response to virus
Innate immune response
Type I interferon signaling pathway
0 5 10 15 20 25
-log10(p-value)
UMAP2
Microglia
UMAP1
0.014 0.012 0.010 0.008 0.006 0.004 0.002 0.000
GO:0060337:Type I interferon signaling pathway
c
P2ry12
Siglech
C4b
Stat1
Isg15
Usp18
Cxcl10
Ddx58
5
0
normalized reads
Rnaset2-/- microglia
Control microglia
d
Oligodendrocytes
OPC
log2 fold change
40 35 30 25 20 15
6 4 2 0 -2
AC149090.1
ROBO1
NPAS3
RNF220
FNBP1
PEX5L
PRR5L
PLP1
ST6GALNAC3
PLCL1
SGK1
OTUD7A
APOD
SPOCK1
SGK3
C4B
IFI27
H2-K1
IFI27L2A
B2M
H2-D1
DDX60
AC149090.1
H2-D1
H2-K1
B2M
STAT1
DDX60
e
VGlut2
Pvalb
Gad1
Gad2
Neuronal Cluster: 1 2 3 4
f
neuronal cluster 2-4 -log10(p-value)
10 5 0 -5 -10 -15
Synaptic signaling
Chemical synaptic transmission
Response to interferon-beta
Defense response

Supplementary Fig. 9 | Single nuclei RNA sequencing analyses of the hippocampus. a, UMAP clustering of the hippocampus from *Rnaset2*$^{-/-}$ and control mice revealed major changes in microglia and neuronal cluster 2. **b,** Upper panel: Differential gene-expression was performed to compare *Rnaset2*$^{-/-}$ and control mice across all detected cell types. GO-term analyses (biological processes) reveals response to virus, innate immune response and type I interferon signaling as the major biological processes to be affected in the hippocampus from *Rnaset2*$^{-/-}$ mice. Lower panel: UMAP indicating that the expression of the type I interferon signaling pathway (GO:0060337) is particularly obvious in the microglia/monocytes cluster. **c,** Violin plot showing the expression of homeostatic microglia marker genes (P2ry12 and Siglec-h), the expression of the reactive microglia marker C4b and some interferon-stimulated genes (Isg15, Usp18, Cxcl10 and Ddx58) in hippocampal microglia cells from *Rnaset2*$^{-/-}$ and control mice. **d,** Analyses of differential gene expression for oligodendrocytes and OPC from *Rnaset2*$^{-/-}$ and control mice reveals only a few differentially expressed genes. **e,** Violin plots indicating the expression of marker genes within the 4 neuronal clusters of hippocampus. The major changes between *Rnaset2*$^{-/-}$ and control mice were obvious by loss of neurons in cluster 2 representing GABAergic inhibitory neurons (Gad1 and Gad2). **f,** Differential gene-expression was performed to compare neuronal clusters 2-4 from *Rnaset2*$^{-/-}$ and control mice. Gene ontology (GO)-term analyses (biological processes) reveals dysregulation of processes linked to neuronal synaptic plasticity genes and upregulation of inflammatory processes.

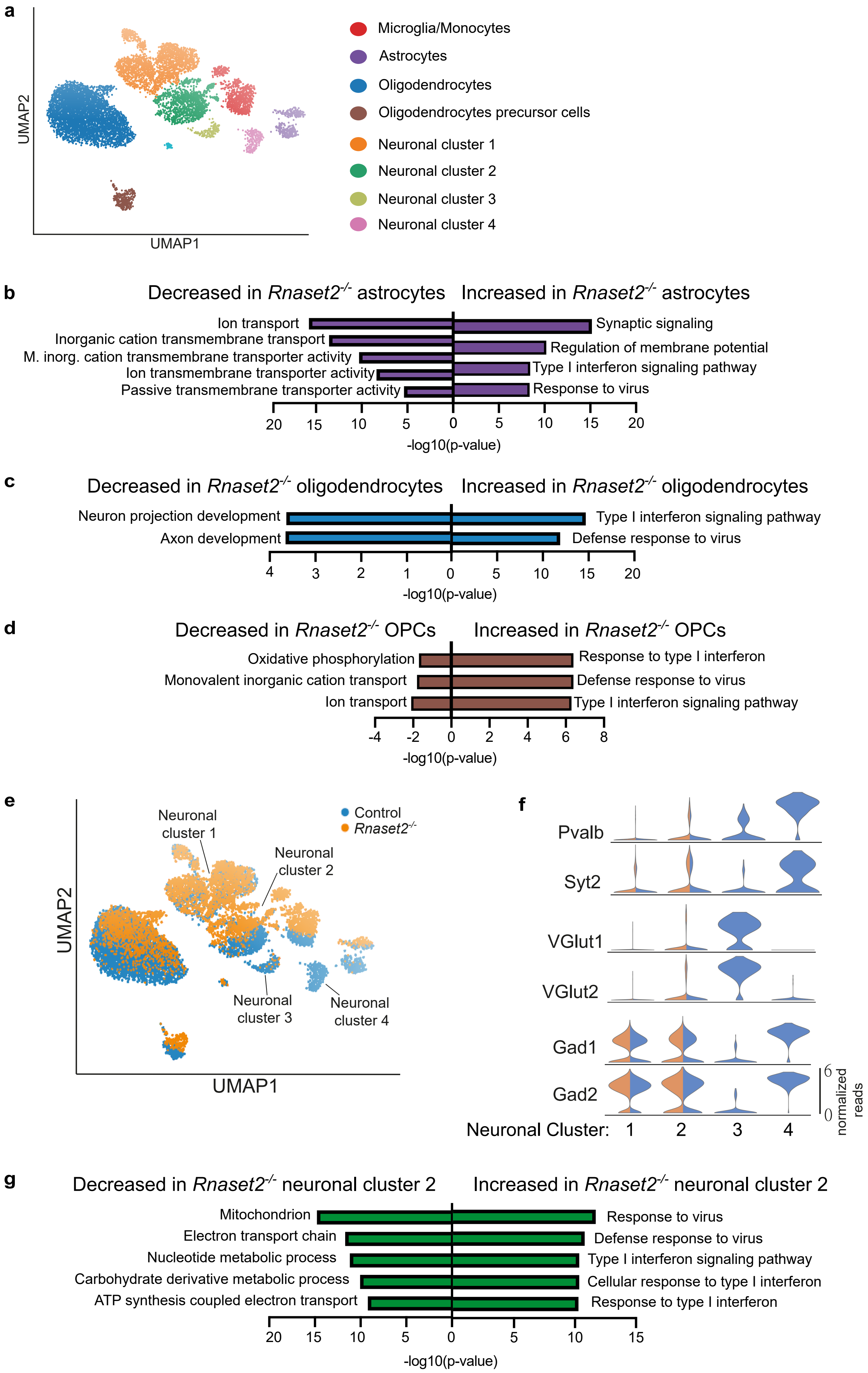
a
UMAP2
UMAP1
Microglia/Monocytes
Astrocytes
Oligodendrocytes
Oligodendrocytes precursor cells
Neuronal cluster 1
Neuronal cluster 2
Neuronal cluster 3
Neuronal cluster 4
b
Decreased in Rnaset2-/- astrocytes
Increased in Rnaset2-/- astrocytes
Ion transport
Inorganic cation transmembrane transport
M. inorg. cation transmembrane transporter activity
Ion transmembrane transporter activity
Passive transmembrane transporter activity
Synaptic signaling
Regulation of membrane potential
Type I interferon signaling pathway
Response to virus
20 15 10 5 0 5 10 15 20
-log10(p-value)
c
Decreased in Rnaset2-/- oligodendrocytes
Increased in Rnaset2-/- oligodendrocytes
Neuron projection development
Axon development
Type I interferon signaling pathway
Defense response to virus
4 3 2 1 0 5 10 15 20
-log10(p-value)
d
Decreased in Rnaset2-/- OPCs
Increased in Rnaset2-/- OPCs
Oxidative phosphorylation
Monovalent inorganic cation transport
Ion transport
Response to type I interferon
Defense response to virus
Type I interferon signaling pathway
-4 -2 0 2 4 6 8
-log10(p-value)
e
Control
Rnaset2-/-
Neuronal cluster 1
Neuronal cluster 2
Neuronal cluster 3
Neuronal cluster 4
UMAP2
UMAP1
f
Pvalb
Syt2
VGlut1
VGlut2
Gad1
Gad2
6
0
normalized reads
Neuronal Cluster: 1 2 3 4
g
Decreased in Rnaset2-/- neuronal cluster 2
Increased in Rnaset2-/- neuronal cluster 2
Mitochondrion
Electron transport chain
Nucleotide metabolic process
Carbohydrate derivative metabolic process
ATP synthesis coupled electron transport
Response to virus
Defense response to virus
Type I interferon signaling pathway
Cellular response to type I interferon
Response to type I interferon
20 15 10 5 0 5 10 15
-log10(p-value)

Supplementary Fig. 10 | Mechanistic insights into interferon-driven neurodegeneration by single nuclei RNA sequencing analyses of the caudate putamen. a, UMAP clustering of the caudate putamen of *Rnaset2*$^{-/-}$ and control mice indicating different cell clusters. **b,** Differential gene expression analyses was performed to compare astrocytes from *Rnaset2*$^{-/-}$ and control mice. Gene ontology (GO)-term analyses (biological processes) reveals dysregulation of processes linked to neuronal support function and inflammatory processes **c,** Differential gene expression analyses was performed to compare oligodendrocytes from *Rnaset2*$^{-/-}$ and control mice. GO-term analyses (biological processes) reveals down-regulation of processes linked to neuronal support function and an upregulation of immune processes including type I interferon signaling in *Rnaset2*$^{-/-}$ mice **d,** Differential gene expression was performed to compare OPCs from *Rnaset2*$^{-/-}$ and control mice. GO-term analyses (biological processes) reveals upregulation of immune processes including type I interferon signaling in *Rnaset2*$^{-/-}$ mice while energy metabolism is reduced **e**, UMAP clustering of the caudate putamen from *Rnaset2*$^{-/-}$ and control mice. **f,** Violin plots indicating the expression of marker genes within the 4 neuronal clusters, suggesting that cluster 4 represents Pvalb positive inhibitory neurons, while cluster 3 expresses marker genes of glutamatergic excitatory neurons. Clusters 1 and 2 expressed marker genes of GABAergic inhibitory neurons. **g,** Differential gene expression was performed to compare neuronal cluster 2 from *Rnaset2*$^{-/-}$ and control mice. GO-term analyses (biological processes) reveals down-regulation of processes linked to energy metabolism and an upregulation of immune processes including type I interferon signaling in *Rnaset2*$^{-/-}$ mice.

Caudate putamen

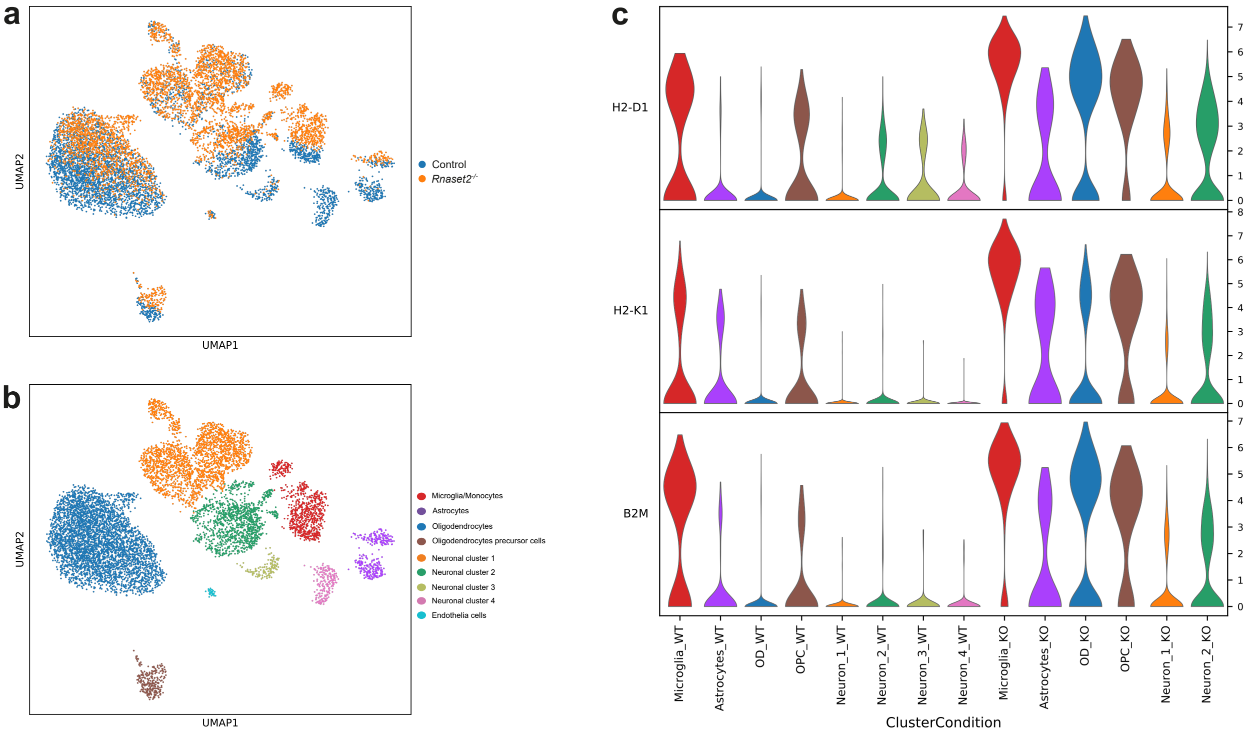

Hippocampus

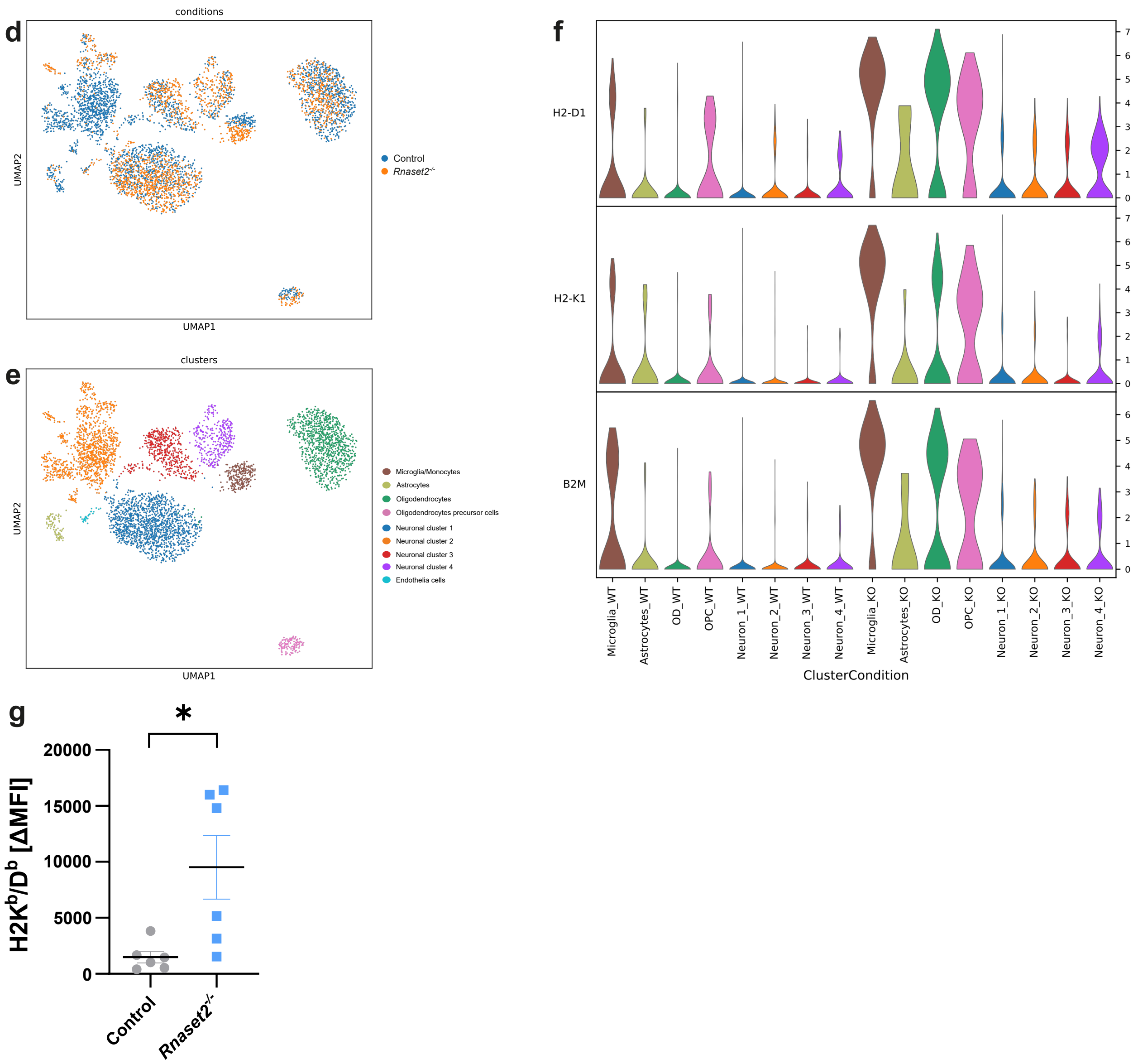

Supplementary Fig. 11 | Analyses of major histocompatibility complex (MHC) class I genes in different CNS cells. UMAP clustering of the caudate putamen from *Rnaset2*$^{-/-}$ and control mice indicating different genotype conditions (**a**) and different cell clusters (**b**). **c,** Violine plot showing the expression of MHC class I genes (*H2-D1*, *H2-K1* and *B2M*) in different cell types and genotypes in caudate putamen. UMAP clustering of the hippocampus from *Rnaset2*$^{-/-}$ and control mice indicating different genotype conditions (**d**) and different cell clusters (**e**). **f,** Violine plot showing the expression of MHC class I genes (*H2-D1*, *H2-K1* and *B2M*) in different cell types and genotypes in hippocampus. **g,** Surface expression of MHC I on microglia cells of *Rnaset2*$^{-/-}$ and control mice (n=6 animals, respectively) assessed by flow cytometry in two independent experiments. The MFI of the isotype control antibody was subtracted from the MFI of the H2Kb/Db staining (ΔMFI). *P* value of two-tailed Welsh's t-test is 0.0360. Data depicted as the mean ± SEM. *P* values are represented as *p<0.05. Source data are provided as a Source Data file.

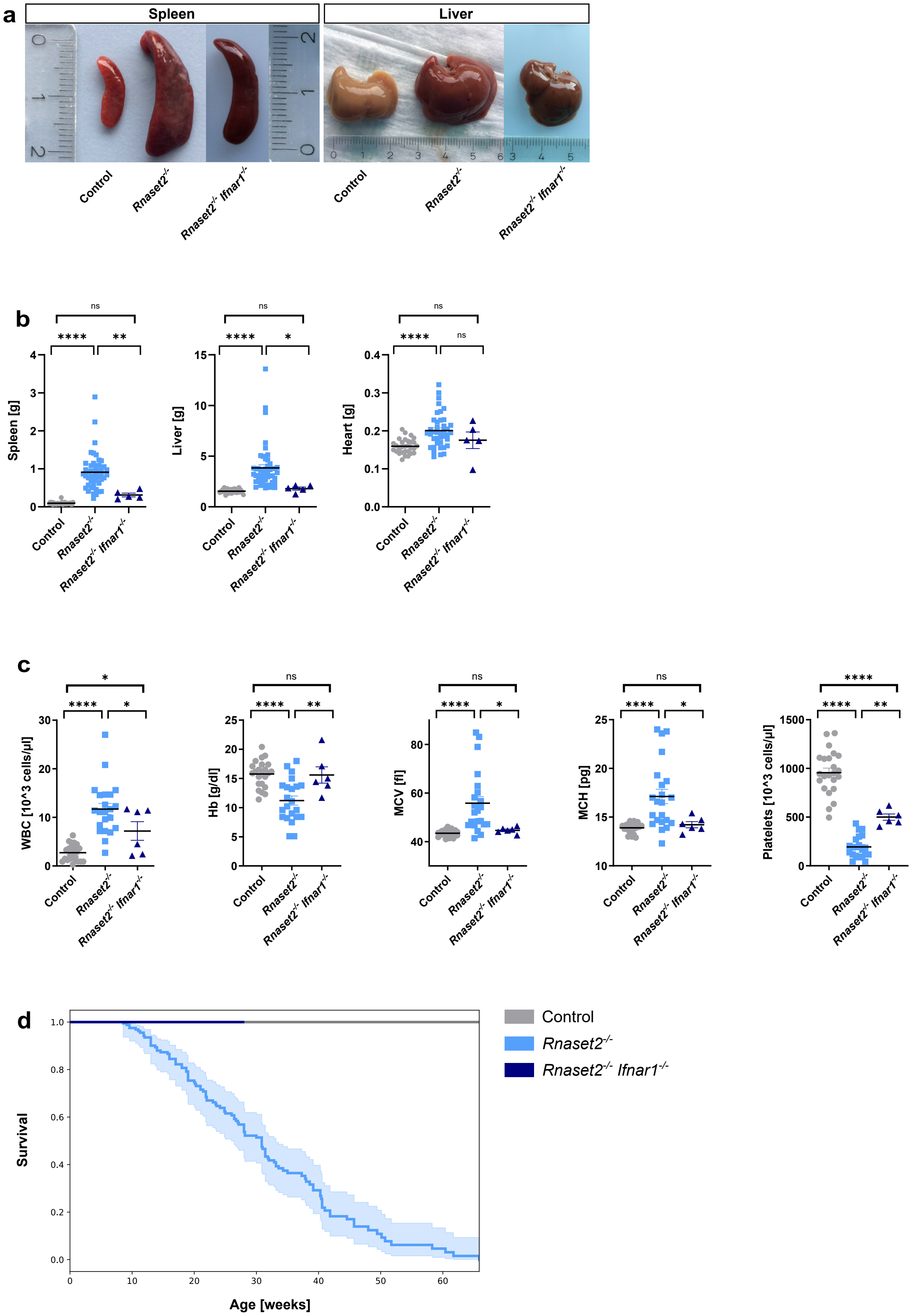
a
Spleen
Liver
Control
Rnaset2-/-
Rnaset2-/- Ifnar1-/-
b
Spleen [g]
Liver [g]
Heart [g]
ns
****
**
*
c
WBC [10^3 cells/µl]
Hb [g/dl]
MCV [fl]
MCH [pg]
Platelets [10^3 cells/µl]
d
Survival
Age [weeks]
Control
Rnaset2-/-
Rnaset2-/- Ifnar1-/-

Supplementary Fig. 12 | Reduced organ size and improved blood values in IFNAR1-deficient *Rnaset2*$^{-/-}$ mice is accompanied by improved survival. a, Representative pictures of spleen and liver from 5-month-old wildtype (control), *Rnaset2*$^{-/-}$ and *Rnaset2*$^{-/-}$ *Ifnar1*$^{-/-}$ mice. **b**, Comparison of organ weights from *Rnaset2*$^{-/-}$ *Ifnar1*$^{-/-}$ (n=5), control (n=30) and *Rnaset2*$^{-/-}$ (n=47 for spleen and liver and n=40 for heart). *Rnaset2*$^{-/-}$ *Ifnar1*$^{-/-}$ mice revealed a significantly milder enlargement of spleen (*p*=0.0025) and liver (*p*=0.0305) compared to *Rnaset2*$^{-/-}$ while heart size was not significantly altered (*p*=0.2763). **c**, Blood counts of *Rnaset2*$^{-/-}$ *Ifnar1*$^{-/-}$ mice (n=6) compared to *Rnaset2*$^{-/-}$ (n=20-21) and control (n=20-21) mice demonstrate a complete rescue of leukocytosis (WBC) and macrocytic (MCV) hyperchromic (MCH) anemia (Hb), while the thrombocytopenia (platelets) is only partially restored. Exact *p* values are provided in a Source Data file. **d**, Survival for *Rnaset2*$^{-/-}$ (blue, n=173) compared with control (grey, n=102) and *Rnaset2*$^{-/-}$ *Ifnar1*$^{-/-}$ (dark blue, n=25) mice visualized as a Kaplan-Meier Curves. *Rnaset2*$^{-/-}$ survival is significantly reduced compared to both control (*p*<0.001) and *Rnaset2*$^{-/-}$ *Ifnar1*$^{-/-}$ (*p*=0.0123). For the survival curve a confidence interval of 95% is estimated using the exponential Greenwood method. The alpha level was corrected using Bonferroni's method by the number of comparisons (alpha=0.05/3). Data depicted as mean ± SEM. *P* values of one-way ANOVA followed by Tukey´s multiple comparison test are represented as **p*<0.05, ***p*<0.01, ****p*<0.001, *****p*<0.0001 and ns *p*≥0.05. For all panels in this figure the majority of values for control and *Rnaset2*$^{-/-}$ mice are also depicted in other figures of this manuscript. Source data are provided as a Source Data file.

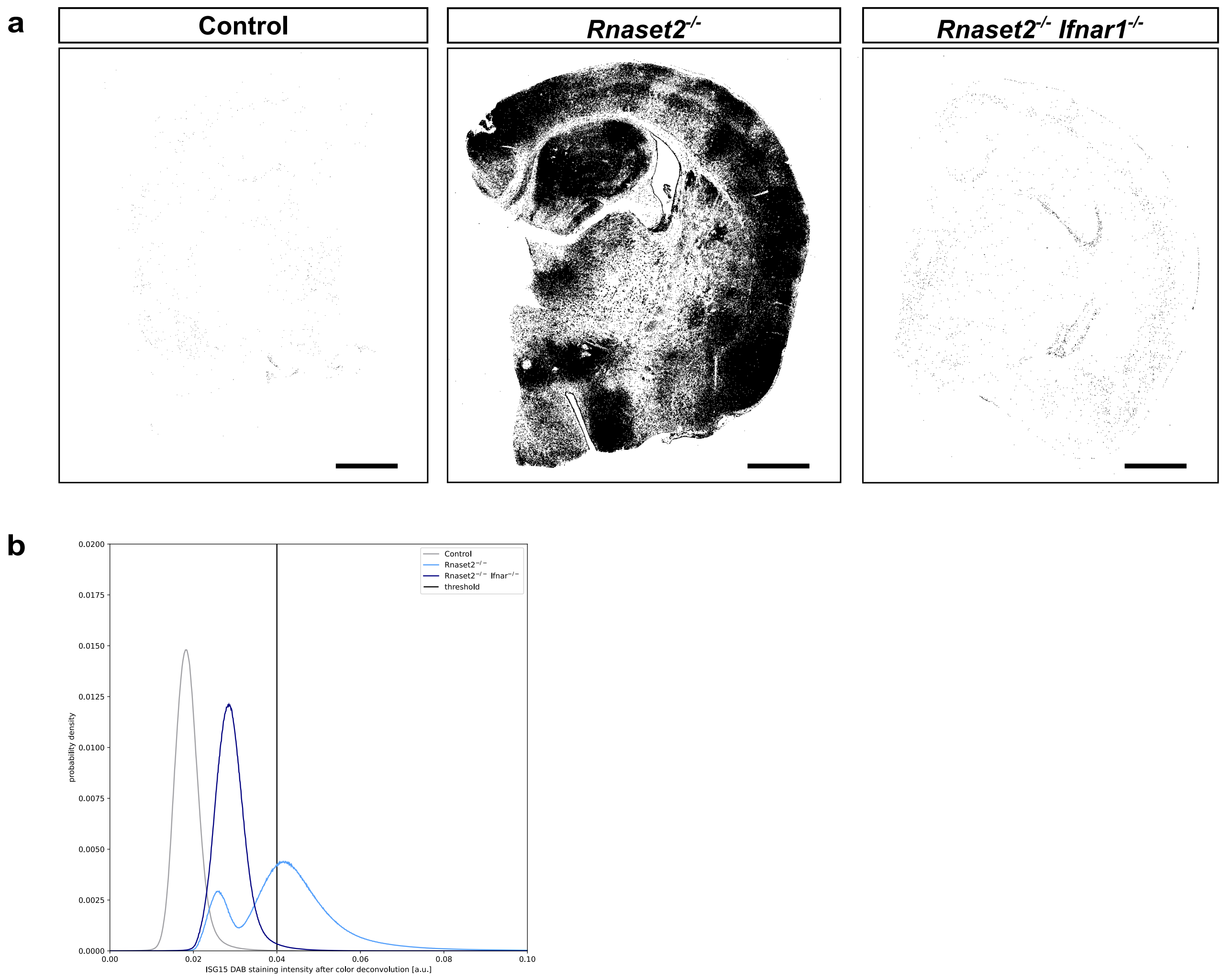

Supplementary Fig. 13 | Color deconvolution of the DAB-ISG15 signal of whole brain hemispheres. a, Corresponding to figure 8b thresholded DAB channel after color deconvolution for ISG15 from whole slide scans (> 20.000 x 20.000 pixels, downsampled display). *Rnaset2*$^{-/-}$*Ifnar1*$^{-/-}$ (n=6) compared to control (n=5) and *Rnaset2*$^{-/-}$ (n=5). Scale bar, 1 mm. **b,** Deconvolved DAB-ISG15 staining intensity histogram of three half cerebral slices for each condition. Source data are provided as a Source Data file.

Supplementary Table 1 | Phenotype comparison of type I interferonopathy mouse models

Gene	Model/Mutation	Genetic background	ISGs	Reduced survival	Defect in hematopoesis		inflammatory infiltration	neurological phenotype
					red	white		
Rnaset2a/b	double knock-out (this study)	C57BL/6	+	+	+	-	++	++
Trex1	Gene targeting[27]	C57BL/6	+	+	-	-	++	-
	Trex1 D18N[26,72]	129S6/SvEvTac	+	+	+	-	++	-
Adar1	Gene targeting[24,49]	C57BL/6	+	+++	+	+	-	-
	erythroid-specific deletion[51]	C57BL/6	+	++	+	-	-	-
	hepatocytes specific KO[50]	C57BL/6	+	+	-	-	+	-
Rnaseh2a	No model available							
Rnaseh2b	$Rnaseh2b^{KOF}$ [29] $Rnaseh2b^{E202X}$ [73]	C57BL/6	-	+++	-	-	-	-
	$RnaseH2^{\Delta GFAP}$ [74]		(+)*	-	-	-	-	-
Rnaseh2c	Rnaseh2c -/- [29]	C57BL/6	-	+++	-	-	-	-
Samhd1	Gene targeting[28]	C57BL/6	+	-	-	-	-	-
Ifih1	Ifih1 gs/+[25]	DBA/2J	++	+	-	-	(+)	-
Usp18	$Usp18^{fl/fl}$ x $Cx3cr1^{Cre}$ [23,75]	C57/Bl6	-	-	-	-	-	+**
DnaseII	$Dnase2a^{-/-}$ x $Ifnar1^{-/-}$ [48,76]	C57BL/6	-	-	+++	-	+***	-

* *ex vivo* - under mitogenic conditions

** microglia specific phenotype

***chronic polyarthritis

Supplementary Table 2 | PrimeTime Assays

Gene	Full name	Assay number
Ifi27	interferon alpha inducible protein 27	Mm.PT.58.42659806
Ifi44	interferon induced protein 44	Mm.PT.58.43920337
Ifit1	interferon induced protein with tetratricopeptide repeats 1	Mm.PT.58.32674307
Isg15	Interferon stimulated gene 15	Mm.PT.58.41476392.g
Rsad2	radical S-adenosyl methionine domain containing protein 2	Mm.PT.58.11280480
Siglec1	sialic acid binding Ig like lectin protein 1	Mm.PT.58.16235548
Usp18	ubiquitin specific peptidase 18	Mm.PT.58.28965870
Cxcl10	C-X-C motif chemokine ligand 10	Mm.PT.58.43575827
Irf7	interferon regulatory factor 7	Mm.PT.58.17554317.g
Ifih1	interferon induced protein with helicase C domain 1	Mm.PT.58.15903724
Ddx58	DEXD/H-box helicase 58	Mm.PT.58.43881955
Rplp0	ribosomal protein subunit P0	Mm.PT.58.43894205
Tbp	TATA-box binding protein	Mm.PT.39a.22214839

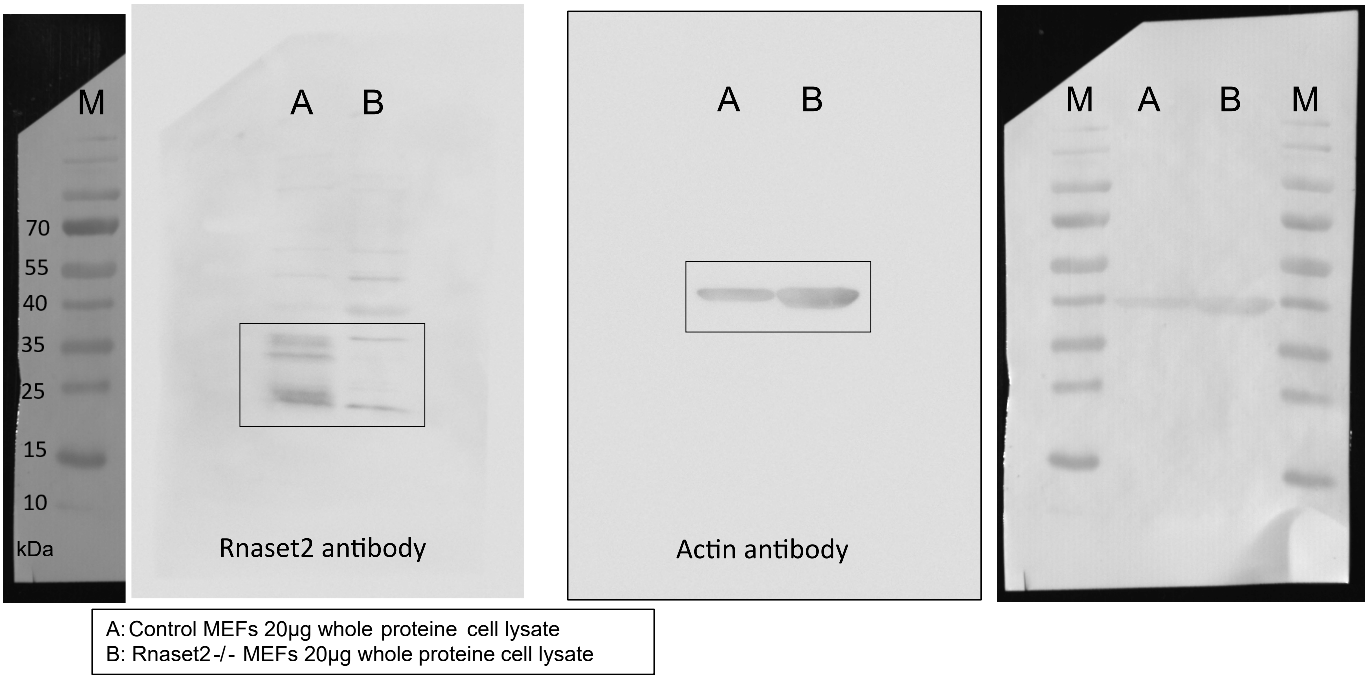

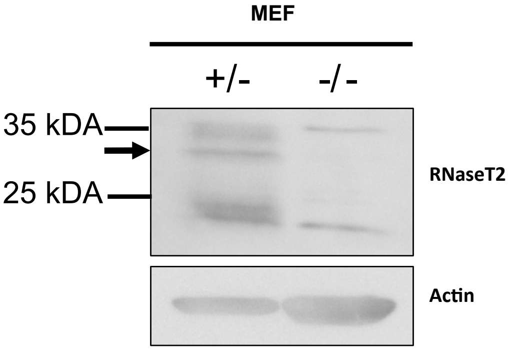

Supplementary Information: Uncropped image of Western Blot from supplementary figure 1b.

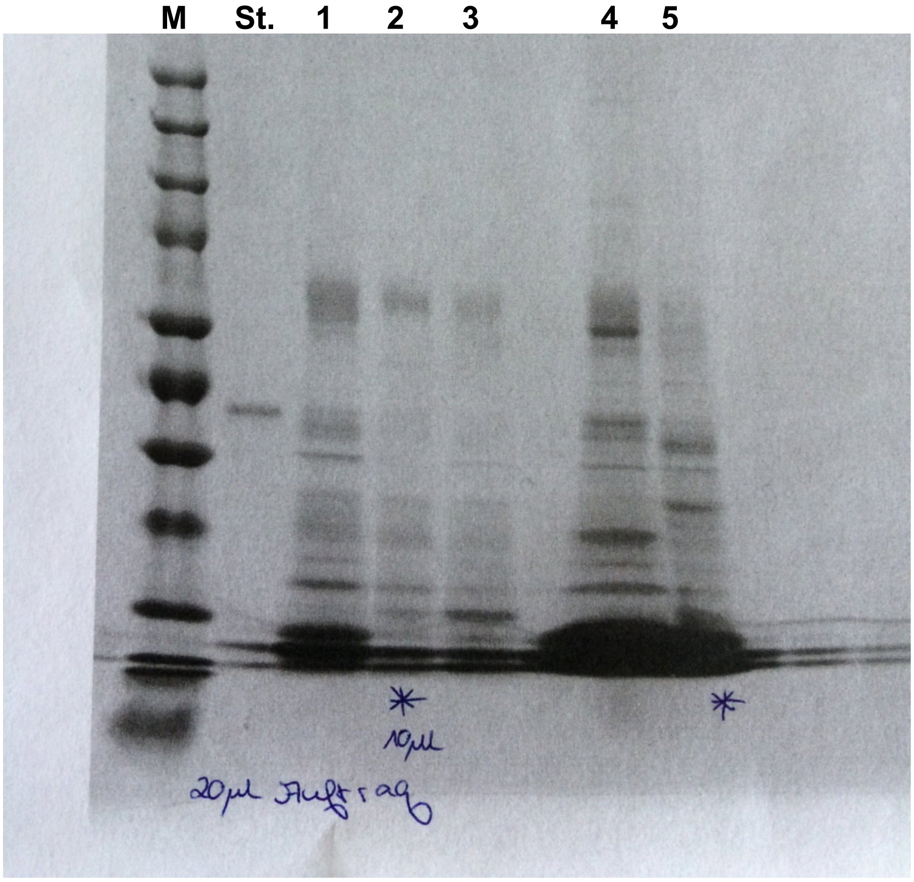

**M**	ProSieve Quad Color (Marker)
**St.**	300 ng Bovine Serum Albumine (BSA) as standard
**1**	Control, female, 20 µl
**2**	Rnaset2-/-, female, 10 µl
**3**	Rnaset2-/-, female, 20 µl
**4**	Control, male, 20 µl
**5**	Rnaset2-/-, male, 10 µl

Supplementary Information: Uncropped image of denaturating Polyacrylamid gel from supplementary figure 4c.
