# Supplementary material for: Interferon-driven brain phenotype in a mouse model of RNaseT2 deficient leukoencephalopathy
Source: Nat Commun. 2021 Nov 11;12:6530. doi: 10.1038/s41467-021-26880-x (PMC8586222; doi:10.1038/s41467-021-26880-x)
Supplement: Supplementary file 3 — Description of Additional Supplementary Files [file 41467_2021_26880_MOESM3_ESM.pdf]

Description of Additional Supplementary Files

Supplementary Data 1: Includes Tables S3-S10

Table S3 - Differentially Expressed genes and GO-term analyses comparing WT vs KO across all cell types of the caudate putamen

Table S4 - Differentially Expressed genes and GO-term analyses comparing WT vs KO in astrocytes of the caudate putamen

Table S5 - Differentially Expressed genes and GO-term analyses comparing WT vs KO in oligodendrocytes of the caudate putamen

Table S6 - Differentially Expressed genes and GO-term analyses comparing WT vs KO in oligodendrocyte precursor cells of the caudate putamen

Table S7 - Differentially Expressed genes and GO-term analyses comparing WT vs KO in neuronal cluster 2 of the caudate putamen

Table S8 - Differentially Expressed genes and GO-term analyses comparing WT vs KO across all cell types of the hippocampus

Table S9 - Differentially Expressed genes and GO-term analyses comparing WT vs KO in microglia of the hippocampus

Table S10 - Differentially Expressed genes and GO-term analyses comparing WT vs KO in neuronal cluster 2-4 of the hippocampus

GO-terms shown in corresponding figure panels are marked yellow and were chosen to reflect representative processes while avoiding listing of redundant processed due similar gene lists
