# Supplementary material for: Interferon-driven brain phenotype in a mouse model of RNaseT2 deficient leukoencephalopathy
Source: Nat Commun. 2021 Nov 11;12:6530. doi: 10.1038/s41467-021-26880-x (PMC8586222; doi:10.1038/s41467-021-26880-x)
Supplement: Supplementary file 5 — Reporting summary [file 41467_2021_26880_MOESM5_ESM.pdf]

nature research

Corresponding author(s): Kettwig, Matthias

Last updated by author(s): Oct 1, 2021

Reporting Summary

Nature Research wishes to improve the reproducibility of the work that we publish. This form provides structure for consistency and transparency in reporting. For further information on Nature Research policies, seeAuthors & Referees and theEditorial Policy Checklist .

Statistics

For all statistical analyses, confirm that the following items are present in the figure legend, table legend, main text, or Methods section.

n/a	Confirmed	
☐	☒	The exact sample size (*n*) for each experimental group/condition, given as a discrete number and unit of measurement
☐	☒	A statement on whether measurements were taken from distinct samples or whether the same sample was measured repeatedly
☐	☒	The statistical test(s) used AND whether they are one- or two-sided<br>*Only common tests should be described solely by name; describe more complex techniques in the Methods section.*
☒	☐	A description of all covariates tested
☐	☒	A description of any assumptions or corrections, such as tests of normality and adjustment for multiple comparisons
☐	☒	A full description of the statistical parameters including central tendency (e.g. means) or other basic estimates (e.g. regression coefficient) AND variation (e.g. standard deviation) or associated estimates of uncertainty (e.g. confidence intervals)
☐	☒	For null hypothesis testing, the test statistic (e.g. *F*, *t*, *r*) with confidence intervals, effect sizes, degrees of freedom and *P* value noted<br>*Give P values as exact values whenever suitable.*
☒	☐	For Bayesian analysis, information on the choice of priors and Markov chain Monte Carlo settings
☒	☐	For hierarchical and complex designs, identification of the appropriate level for tests and full reporting of outcomes
☒	☐	Estimates of effect sizes (e.g. Cohen's *d*, Pearson's *r*), indicating how they were calculated

Our web collection on statistics for biologists contains articles on many of the points above.

Software and code

Policy information about availability of computer code

Data collection	Behavioral testing: ANY-maze v5.1 (Stoelting Co.); FACS: BD FACSDiva Software v8.0.2 (BD Biosciences); qPCR: QuantStudio 3 Real-Time PCR System (Applied Biosystems); Microscopy: Axio Imager.M1 microscope (Zeiss) using 10x/0.3, 20x/0.5 and 40x/0.75 Plan-NEOFLUAR objectives and an Axiocam 105 color camera (Zeiss) or an Olympus BX51 light or combined light and fluorescent microscope; Ex vivo MRI: 300 MHz (7 T) vertical wide bore system, using a transmit/receive birdcage radiofrequency coil with an inner diameter of 25 mm and a 1 Tm−1 gradient insert (Bruker) interfaced to a Linux operating system running Topspin 2.0 and Para Vision 3.0 imaging software (Bruker Biospin); In vivo MRI: 94/30 BioSpec (Bruker BioSpin); Single nuclei RNA sequencing: 10X Chromium Next GEM Single Cell on Illumina NextSeq 550.
Data analysis	GraphPad Prism version 8.3.0 (GraphPad Software Inc, San Diego, CA); Ex vivo MRI: image sequence analysis (ISA) tool package (Paravision 5, Bruker) and OriginPro v. 8 (OriginLab); FACS: FlowJo™ Software Version 10.6.1 (Becton, Dickinson and Company, 2019); qPCR: QuantStudio™ Design & Analysis Software v1.4.3; In vivo MRI: Amira 6.2.0 (Thermo Fisher Scientific) for semiautomatic segmentation; Single nuclei RNA sequencing: CellRanger software v.4.0.0 (10XGenomics), SCANPY package v1.7.2 and ShinyGOVo.60; DAB-ISG15 signal analysis: Scikit-image v0.18.1 inlcuding automatic normalization (for details see public discussion on scikit-image developer page: https://github.com/scikit-image/scikit-image/pull/4725); Survival curves: open source python package lifelines (v0.26.0).

For manuscripts utilizing custom algorithms or software that are central to the research but not yet described in published literature, software must be made available to editors/reviewers. We strongly encourage code deposition in a community repository (e.g. GitHub). See the Nature Research guidelines for submitting code & software for further information.

Data

Policy information about availability of data

All manuscripts must include a data availability statement. This statement should provide the following information, where applicable:

- Accession codes, unique identifiers, or web links for publicly available datasets
- A list of figures that have associated raw data
- A description of any restrictions on data availability

Raw data for all results/figures presented in this article, including Supplementary figures, are available in the Source Data file.
Any additional data are available upon request. Source data are provided with this paper.
Raw single nuclei RNA sequencing data are accessible from the NCBI GEO database using the accession number GSE180138.
https://www.ncbi.nlm.nih.gov/geo/query/acc.cgi?acc=GSE180138
Detailed annotation tables for these datasets are provided in Supplementary Table 3-7.
Mus musculus reference genome (GRCm38.p6) use for single RNA nuclei study is accessible from NCBI GenBank under the accession numbers GCA_000001635.8.
Reference Sequence for Rnaset2a and Rnaset2b gene are accessible from NCBI GenBank under the accession numbers NM_001083938.3 and NM_0266211.2 respectively.

Field-specific reporting

Please select the one below that is the best fit for your research. If you are not sure, read the appropriate sections before making your selection.

☒ Life sciences ☐ Behavioural & social sciences ☐ Ecological, evolutionary & environmental sciences

For a reference copy of the document with all sections, see nature.com/documents/nr-reporting-summary-flat.pdf

Life sciences study design

All studies must disclose on these points even when the disclosure is negative.

Sample size	Sample size was predetermined according to the type of experiment based on common practice.<br>For most mouse experiments 4-8 mice were used and the same number of controls (FACS analysis, histology, bone marrow analysis, immunohistochemistry, MRI, RNA sequencing, qPCR).<br>Blood values (blood count and serum) was taken and analyzed from most animals, which were transcardially perfused for further analysis.<br>Organ weight was performed whenever the further processing of the animals allowed this.<br>Sample size for behavioral analysis was determined by a priori one-way anova (effect thickness: 0.4; Error first type: 0.05; Error second type: 0.2; power 80%).
Data exclusions	No replicates were excluded.
Replication	All experiments were conducted in at least 3 biological replicates and the experimental findings could be confirmed thereby.
Randomization	Randomization was not relevant to the study, because we determine in all experiments the differences between different genotypes.
Blinding	For most examinations the investigators were blinded by using lab codes for each sample not providing the genotype of the animal.<br>For other experiments, the investigators were not blinded to group allocation as they performed both the experiment and analysis; blinding was not possible.

Reporting for specific materials, systems and methods

We require information from authors about some types of materials, experimental systems and methods used in many studies. Here, indicate whether each material, system or method listed is relevant to your study. If you are not sure if a list item applies to your research, read the appropriate section before selecting a response.

Materials & experimental systems

n/a	Involved in the study	
☐	☒	Antibodies
☒	☐	Eukaryotic cell lines
☒	☐	Palaeontology
☐	☒	Animals and other organisms
☒	☐	Human research participants
☒	☐	Clinical data

Methods

n/a	Involved in the study	
☒	☐	ChIP-seq
☐	☒	Flow cytometry
☐	☒	MRI-based neuroimaging

Antibodies

Antibodies used

For immunohistochemistry:

anti-CD41, Abcam, Cat#ab134131, Lot#GR3259786-4, Clone#EPR4330
anti-CD3, Abcam, Cat#ab16669, Lot#GR3285725-6, Clone#SP7
anti-B220, BD Pharmingen, Cat#557390, Lot#8152600, Clone#RA3-6B2
anti-myelin basic protein, Abcam, Cat#ab7349, Lot#GR3221078-15, Clone#12
anti-CD3, DCS, Cat#CI597C01, Lot#W298, Clone#SP7
anti-Mac3, BD Pharmingen, Cat#553322, Lot#6022547, Clone#M3/84
anti CD8a, Invitrogen, Cat#14-0808-82, Lot#04221B, Clone#4SM15
anti-GFAP, Synaptic Systems, Cat#173011, Lot#1-12, Clone#134B1
anti-Iba1, Merck/Millipore, Cat#MABN92, Lot#3712470, Clone#20A12.1
anti-NeuN, Abcam, Cat#ab177487, Lot#GR3275122-4, Clone#EPR12763
anti-TMEM119, Abcam, Cat#ab209064, Lot#GR3211941-16, Clone#28-3
anti-ISG15, kindly provided by Marco Prinz
anti-CD31, Dianova, Cat#DIA-310, Lot#201015/05, Clone#SZ31

For FACS analysis:

BV605 anti-mouse c-kit, Biolegend, Cat#105847, Lot#B274670, Clone#2B8,
AF488 anti-mouse Ter119/erythroid cells, Biolegend, Cat#116215, Lot#B270111, Clone#TER-119
PE/Cy7 anti-mouse CD3ε, Biolegend, Cat#100320, Lot#B239868, Clone#145-2C11
PE/Cy5 anti-mouse CD4, Biolegend, Cat#100513, Lot#B256773, Clone#RM4-5
BV605 anti-mouse CD8α, Biolegend, Cat#100743, Lot#B279615, Clone#53-6.7
PerCP/Cy.5.5 anti-mouse/human CD11b, Biolegend, Cat#101228, Lot#B236435, Clone#M1/70
BV711 anti-mouse CD16/32, Biolegend, Cat#101337, Lot#B269751, Clone#93
BUV395 rat-anti-mouse CD19, BD, Cat#563557, Lot#8206694, Clone#1D3
PE/Cy7 anti-mouse CD34, Biolegend, Cat#128617, Lot#B277974, Clone#HM34
BV510 anti-mouse CD41, Biolegend, Cat#133923, Lot#B258166, Clone#MWReg30,
APC anti-human/mouse CD44, eBioscience, Cat#17-0441-82, Lot#431247, Clone#IM7
BV785 anti-mouse/ human CD44, Biolegend, Cat#103041, Lot#B281256, Clone#IM7
APC/Cy7 anti-mouse CD45, Biolegend, Cat#103116, Lot#B324364, Clone#30F11
BV510 anti mouse CD45R/B220, Biolegend, Cat#103247, Lot#B313156, Clone#RA3-6B2
BUV395 rat anti mouse CD45R/B220, BD, Cat#563793, Lot#9028674, Clone#RA3-6B2
PE anti-mouse CD62L, Biolegend, Cat#104408, Lot#B322380, Clone#MEL-14
APC anti-mouse CD71, Biolegend, Cat#113819, Lot#B264912, Clone#RI7217
Pacific Blue anti-mouse CD105, Biolegend, Cat#120411, Lot#B245561, Clone#MJ7/18
PerCP Cy5.5 anti-mouse CD150, Biolegend, Cat#115921, Lot#B318999, Clone#TC15-12F12.2
BV421 anti-mouse TCRβ chain, Biolegend, Cat#109229, Lot#B264324, Clone#H57-597
FITC anti-mouse gamma delta TCR, eBioscience, Cat#11-5711-82, Lot#E00635-1198, Clone#eBioGL3
PE/Cy5 anti-mouse Ly-6A/6E (Sca-1), Biolegend, Cat#108109, Lot#B253864, Clone#D7
PE/Cy7 anti-mouse Ly-6C, Biolegend, Cat#128018, Lot#B200606, Clone#HK1.4
BV510 anti-mouse Ly6G, Biolegend, Cat#127633, Lot#B336186, Clone#1A8
AF700 anti-mouse Ly6G/Ly-6C (Gr-1), Biolegend, Cat#108421, Lot#B323270, Clone#RB6-8C5
APC anti-mouse CCR2, R&D, Cat#FAB5538A, Lo#ABLE0314121, Clone#475301
Biotin anti-mouse P2RY12; Biolegend, Part#95566, Lot#B263990, Clone#S16007D
PE anti-mouse αH2Kb/Db antibody, Biolegend, Cat#114607, Lot#B327570, Clone#28-8-6
PE mouse IgG2a isotype control, Biolegend, Cat#400213, Lot#B277922, Clone#MOPC-173

For western blotting:

anti-mouse RNaseT2, Cloud-Clone Corp, Cat#RPA113Mu01, Lot#A20191010620
anti-mouse β-actin, Sigma, Cat#A5441, Lot#026M4780V

Validation

anti-CD41, Abcam, Cat#ab134131, Lot#GR3259786-4, Clone#EPR4330, validation stated on supplier's website: https://www.abcam.com/cd41-antibody-epr4330-ab134131.html
anti-CD3, Abcam, Cat#ab16669, Lot#GR3285725-6, Clone#SP7, validation stated on supplier's website: https://www.abcam.com/cd3-antibody-sp7-ab16669.html
anti-B220, BD Pharmingen, Cat#557390, Lot#8152600, Clone#RA3-6B2, validation stated on supplier's website: https://www.bdbiosciences.com/en-us/products/reagents/flow-cytometry-reagents/research-reagents/single-color-antibodies-ruo/apc-rat-anti-mouse-cd45r-b220.553092
anti-myelin basic protein, Abcam, Cat#ab7349, Lot#GR3221078-15, Clone#12, validation stated on supplier's website: https://www.abcam.com/myelin-basic-protein-antibody-12-ab7349.html

anti-CD3, DCS, Cat#CI597C01, Lot#W298, Clone#SP7, validation stated on supplier's website: http://s760772780.online.de/index.php?id=114&item_selectors=item_name%23clone%23isotype%23producer&item_specifire=CD3%23SP7%23Kaninchen%20IgG

anti-Mac3, BD Pharmingen, Cat#553322, Lot#6022547, Clone#M3/84, validation stated on supplier's website: https://www.bdbiosciences.com/en-us/products/reagents/flow-cytometry-reagents/research-reagents/single-color-antibodies-ruo/purified-rat-anti-mouse-cd107b.553322

anti CD8a, Invitrogen, Cat#14-0808-82, Lot#04221B, Clone#4SM15, validation stated on supplier's website: https://www.thermofisher.com/antibody/product/CD8a-Antibody-clone-4SM15-Monoclonal/14-0808-82

anti-GFAP, Synaptic Systems, Cat#173011, Lot#1-12, Clone#134B1, validation stated on supplier's website: https://www.sysy.com/product/173011

anti-Iba1, Merck/Millipore, Cat#MABN92, Lot#3712470, Clone#20A12.1, validation stated on supplier's website: https://www.merckmillipore.com/DE/de/product/Anti-Iba1-AIF1-Antibody,MM_NF-MABN92

anti-NeuN, Abcam, Cat#ab177487, Lot#GR3275122-4, Clone#EPR12763, validation stated on supplier's website: https://www.abcam.com/neun-antibody-epr12763-neuronal-marker-ab177487.html

anti-TMEM119, Abcam, Cat#ab209064, Lot#GR3211941-16, Clone#28-3, validation stated on supplier's website: https://www.abcam.com/tmem119-antibody-28-3-microglial-marker-ab209064.html?productWallTab=ShowAll

anti-ISG15, kindly provided by Marco Prinz, validation stated in Osiak, A.; Utermöhlen, O.; Niendorf, S.; Horak, I.; Knobeloch, K.-P. ISG15, an interferon-stimulated ubiquitin-like protein, is not essential for STAT1 signaling and responses against vesicular stomatitis and lymphocytic choriomeningitis virus. Molecular and cellular biology [Online] 2005, 25 (15), 6338–6345.

anti-CD31, Dianova, Cat#DIA-310, Lot#201015/05, Clone#SZ31, validation stated on supplier's website: https://www.dianova.com/shop/dia-310-anti-cd31-mssw-aus-ratte-sz31-unkonj-fur-paraffingewebe-aus-maus/

BV605 anti-mouse c-kit, Biolegend, Cat#105847, Lot#B274670, Clone#2B8, validation stated on supplier's website: https://www.biolegend.com/de-de/products/brilliant-violet-605-anti-mouse-cd117-c-kit-antibody-16969

AF488 anti-mouse Ter119/erythroid cells, Biolegend, Cat#116215, Lot#B270111, Clone# TER-119, validation stated on supplier's website: https://www.biolegend.com/de-de/products/alexa-fluor-488-anti-mouse-ter-119-erythroid-cells-antibody-2714

PE/Cy7 anti-mouse CD3ε, Biolegend, Cat#100320, Lot#B239868, Clone#145-2C11, validation stated on supplier's website: https://www.biolegend.com/de-de/products/pe-cyanine7-anti-mouse-cd3epsilon-antibody-1899

PE/Cy5 anti-mouse CD4, Biolegend, Cat#100513, Lot#B256773, Clone#RM4-5, validation stated on supplier's website: https://www.biolegend.com/de-de/products/pe-cyanine5-anti-mouse-cd4-antibody-483

BV605 anti-mouse CD8α, Biolegend, Cat#100743, Lot#B279615, Clone#53-6.7, validation stated on supplier's website: https://www.biolegend.com/de-de/products/brilliant-violet-605-anti-mouse-cd8a-antibody-7636

PerCP/Cy.5.5 anti-mouse/human CD11b, Biolegend, Cat#101228, Lot#B236435, Clone# M1/70, validation stated on supplier's website: https://www.biolegend.com/de-de/products/percp-cyanine5-5-anti-mouse-human-cd11b-antibody-4257

BV711 anti-mouse CD16/32, Biolegend, Cat#101337, Lot#B269751, Clone#93, validation stated on supplier's website: https://www.biolegend.com/de-de/products/brilliant-violet-711-anti-mouse-cd16-32-11835

BUV395 rat-anti-mouse CD19, BD, Cat#563557, Lot#8206694, Clone#1D3, validation stated on supplier's website: https://www.bdbiosciences.com/en-in/products/reagents/flow-cytometry-reagents/research-reagents/single-color-antibodies-ruo/buv395-rat-anti-mouse-cd19.563557

PE/Cy7 anti-mouse CD34, Biolegend, Cat#128617, Lot#B277974, Clone#HM34, validation stated on supplier's website: https://www.biolegend.com/de-de/products/pe-cyanine7-anti-mouse-cd34-antibody-15031

BV510 anti-mouse CD41, Biolegend, Cat#133923, Lot#B258166, Clone#MWReg30, validation stated on supplier's website: https://www.biolegend.com/de-de/products/brilliant-violet-510-anti-mouse-cd41-antibody-10072

APC anti-human/mouse CD44, eBioscience, Cat#17-0441-82, Lot#431247, Clone#IM7, validation stated on supplier's website: https://www.thermofisher.com/antibody/product/ CD44-Antibody-clone-IM7-Monoclonal/17-0441-82

BV785 anti-mouse/ human CD44, Biolegend, Cat#103041, Lot#B281256, Clone#IM7, validation stated on supplier's website: https://www.biolegend.com/de-de/products/brilliant-violet-785-anti-mouse-human-cd44-antibody-7959

APC/Cy7 anti-mouse CD45, Biolegend, Cat#103116, Lot#B324364, Clone#30F11, validation stated on supplier's website: https://www.biolegend.com/de-de/products/apc-cyanine7-anti-mouse-cd45-antibody-2530

BV510 anti mouse CD45R/B220, Biolegend, Cat#103247, Lot#B313156, Clone#RA3-6B2, validation stated on supplier's website: https://www.biolegend.com/de-de/products/brilliant-violet-510-anti-mouse-human-cd45r-b220-antibody-7996

BUV395 rat anti mouse CD45R/B220, BD, Cat#563793, Lot#9028674, Clone#RA3-6B2, validation stated on supplier's website: https://www.bdbiosciences.com/en-eu/products/reagents/flow-cytometry-reagents/research-reagents/single-color-antibodies-ruo/buv395-rat-anti-mouse-cd45r-b220.563793

PE anti-mouse CD62L, Biolegend, Cat#104408, Lot#B322380, Clone#MEL-14, validation stated on supplier's website: https://www.biolegend.com/de-de/products/pe-anti-mouse-cd62l-antibody-386

APC anti-mouse CD71, Biolegend, Cat#113819, Lot#B264912, Clone#RI7217, validation stated on supplier's website: https://www.biolegend.com/de-de/products/apc-anti-mouse-cd71-antibody-15498

Pacific Blue anti-mouse CD105, Biolegend, Cat#120411, Lot#B245561, Clone#MJ7/18, validation stated on supplier's website: https://www.biolegend.com/de-de/products/pacific-blue-anti-mouse-cd105-antibody-6080

PerCP Cy5.5 anti-mouse CD150, Biolegend, Cat#115921, Lot#B318999, Clone#TC15-12F12.2, validation stated on supplier's website: https://www.biolegend.com/de-de/products/percp-cyanine5-5-anti-mouse-cd150-slam-antibody-4290

BV421 anti-mouse TCRβ chain, Biolegend, Cat#109229, Lot#B264324, Clone#H57-597, validation stated on supplier's website: https://www.biolegend.com/de-de/products/brilliant-violet-421-anti-mouse-tcr-beta-chain-antibody-7251

FITC anti-mouse gamma delta TCR, eBioscience, Cat#11-5711-82, Lot#E00635-1198, Clone#eBioGL3, validation stated on supplier's website: https://www.thermofisher.com/ antibody/product/TCR-gamma-delta-Antibody-clone-eBioGL3-GL-3-GL3-Monoclonal/11-5711-82

PE/Cy5 anti-mouse Ly-6A/6E (Sca-1), Biolegend, Cat#108109, Lot#B253864, Clone#D7, validation stated on supplier's website: https://www.biolegend.com/de-de/products/pe-cyanine5-anti-mouse-ly-6a-e-sca-1-antibody-229

PE/Cy7 anti-mouse Ly-6C, Biolegend, Cat#128018, Lot#B200606, Clone#HK1.4, validation stated on supplier's website: https://www.biolegend.com/de-de/products/pe-cyanine7-anti-mouse-ly-6c-antibody-6063

BV510 anti-mouse Ly6G, Biolegend, Cat#127633, Lot#B336186, Clone#1A8, validation stated on supplier's website: https://www.biolegend.com/de-de/products/brilliant-violet-510-anti-mouse-ly-6g-antibody-9121
AF700 anti-mouse Ly6G/Ly-6C (Gr-1), Biolegend, Cat#108421, Lot#B323270, Clone#RB6-8C5, validation stated on supplier's website: https://www.biolegend.com/de-de/products/alexa-fluor-700-anti-mouse-ly-6g-ly-6c-gr-1-antibody-3390
APC anti-mouse CCR2, R&D, Cat#FAB5538A, Lo#ABLE0314121, Clone#475301, validation stated on supplier's website: https://www.rndsystems.com/products/mouse-ccr2-apc-conjugated-antibody-475301_fab5538a
Biotin anti-mouse P2RY12; Biolegend, Part#95566, Lot#B263990, Clone#S16007D, validation stated on supplier's website: https://www.biolegend.com/de-de/products/biotin-anti-p2ry12-antibody-16149
PE anti-mouse αH2Kb/Db antibody, Biolegend, Cat#114607, Lot#B327570, Clone#28-8-6; validation stated on supplier's website: https://www.biolegend.com/de-de/products/pe-anti-mouse-h-2k-b-h-2d-b-antibody-1686
PE mouse IgG2a isotype control, Biolegend, Cat#400213, Lot#B277922, Clone#MOPC-173; validation stated on supplier's website: https://www.biolegend.com/de-de/products/pe-mouse-igg2a-kappa-isotype-ctrl-fc-3043
anti-mouse RNaseT2, Cloud-Clone Corp, Cat#RPA113Mu01, Lot#A20191010620, validation stated on supplier's website: http://www.cloud-clone.com/products/RPA113Mu01.html
anti-mouse β-actin, Sigma-Aldrich, Cat#A5441, Lot#026M4780V, validation stated on supplier's website: https://www.sigmaaldrich.com/DE/en/product/sigma/a5441

Animals and other organisms

Policy information about studies involving animals; ARRIVE guidelines recommended for reporting animal research

Laboratory animals	This study involved Rnaset2-/- mice (mus musculus) bred on a C57BL/6N genetic background between 2 to 10 months of mixed gender. Heterozygous littermattes and wildtype animals were used as controls.<br>Further animals used in this study are Ifnar1-/- mice (mus musculus) crossbred with Rnaset2-/- mice. 6 male und 1 female of double knockout mice Rnaset2-/- Ifnar1-/- with 11 weeks and 4-6 month are analyzed with littermates as controls.
Wild animals	This study did not include wild animals.
Field-collected samples	This study did not include field-collected samples.
Ethics oversight	All experiments were performed in accordance with the German animal protection law and with the permission of the Lower Saxony Federal State Office for Consumer Protection and Food Safety (LAVES) under protocol No. 17-2697.

Note that full information on the approval of the study protocol must also be provided in the manuscript.

Flow Cytometry

Plots

Confirm that:

- [x] The axis labels state the marker and fluorochrome used (e.g. CD4-FITC).
- [x] The axis scales are clearly visible. Include numbers along axes only for bottom left plot of group (a 'group' is an analysis of identical markers).
- [x] All plots are contour plots with outliers or pseudocolor plots.
- [x] A numerical value for number of cells or percentage (with statistics) is provided.

Methodology

Sample preparation	For spleen: Single-cell suspensions were prepared by mashing spleen through a 70 µm cell strainer (Greiner Bio-One). Cells were washed with FACS buffer (PBS +2 % FBS) and incubated for 15 min in Fc-blocking buffer and stained with corresponding anti-mouse antibodies for 30 minutes on ice protected from light.<br>For CNS: Brain and spinal cord were removed and dissociated with the gentle MACSTM dissociator. The tissue was digested for 35 min with 2,5 mg/ml collagenase D (Roche) and 1 mg/ml DNaseI (Roche) at 37°C. Mononuclear cells were isolated by Percoll gradient centrifugation (37 % / 70 %, GE Healthcare) removed from the interphase, washed and subsequently blocked with αCD16/32 (BioLegend, Clone 93) for 15 min.
Instrument	BD LSRFortessaTM X-20 (BD Biosciences) or FACS CantoTM II (BD Biosciences)
Software	Data were collected with BD FACSDiva Software Version 8.0.1 and analyzed in FlowJo™ Software Version 10.6.1 (Becton, Dickinson and Company, 2019)
Cell population abundance	As we use no cell-sorting in our study this is not relevant.
Gating strategy	Gating strategies are described in the manuscript. More detailed information will be provided upon request.

- [x] Tick this box to confirm that a figure exemplifying the gating strategy is provided in the Supplementary Information.

Magnetic resonance imaging

Experimental design

Design type	No functional MRI (fMRI) data were acquired.
Design specifications	As we use structural MRI data this is not relevant.
Behavioral performance measures	As we use structural MRI data this is not relevant.

Acquisition

Imaging type(s)	Ex-vivo: structural and relaxometry analysis<br>In-vivo: structural and perfusion analysis
Field strength	Ex-vivo: 7.0 Tesla<br>In-vivo: 9.4 Tesla
Sequence & imaging parameters	Ex-vivo:<br>Rapid acquisition T2-weighted MR images with relaxation enhancement (RARE) sequence. Effective echo time= 18.1 ms; repetition time= 5 s; flip angle= 90°; averages= 32 (for sagittal and axial) or 64 (for coronal); and RARE factor (echo train length)= 4; image slice thickness= 0.5 mm. The field of view was 2.5 × 2.5 cm2, with an image matrix of 256 × 256. This yields an effective in-plane resolution of approximately 97 µm.<br>Multi-slice multi-echo (MSME) sequence for transverse relaxation time (T2) measurement based on the Carr-Purcell Meiboom-Gill (CPMG) sequence, where transverse magnetization of a 90º pulse is refocused by a train of 180º pulses generating a series of echoes. Nominal flip angles= 90° and 180°, and a train of 12 echoes with TEs ranging from 8.17 ms to 98 ms with 8.17 ms echo-spacing; TR= 2 s, slice thickness= 0.5 mm; number of slices 8 and a matrix size 256 x 256 pixels.<br><br>In vivo:<br>Multi-slice T2-weighted images* (RARE, TR/effective TE= 6843/55 ms, number of echoes 8) were obtained with a special resolution of 40 x 40 x 300 µm3.<br>For dynamic contrast enhanced MRI (DCM) a 3D T1-weighted data set (FLASH, TR/TE= 18/3.4, flip angle= 8°, 100 µm isotropic spatial resolution) was acquired before (S0) and 20 minutes after (SC) intravenous injection (via tail vein catheter) of 0.3 mmol/Kg body weight Gd-DTPA (Magnevist®, Bayer Vital GmbH, diluted to 0.1 mM in physiological saline)
Area of acquisition	Whole brain scan

Diffusion MRI ☐ Used ☒ Not used

Preprocessing

Preprocessing software	Ex-vivo: Linux operating system running Topspin 2.0 and Para Vision 3.0 imaging software (Bruker Biospin).<br>In-vivo: Software package Amira 6.2.0 (Thermo Fisher Scientific) for semiautomatic segmentation.
Normalization	Magnetic field homogeneity was optimized by shimming
Normalization template	Each session of measurements started with a multi-slice orthogonal gradient-echo sequence for position determination and selection of the desired region for subsequent experiments.
Noise and artifact removal	Magnetic field homogeneity was optimized by shimming
Volume censoring	For measurement of brain areas, the desired region of interest was drawn on the image and areas were computed using an image sequence analysis (ISA) tool package (Paravision 5, Bruker). The data were exported to OriginPro v. 8 (OriginLab) for further analysis and percentage of specific area with respect to whole brain area was calculated.

Statistical modeling & inference

Model type and settings	For calculation of T2 relaxation time, regions of interest (ROIs) were drawn at desired locations within the brain using an image sequence analysis (ISA) tool package (Paravision 5, Bruker). Monoexponential fitting was then used to calculate T2 using a monoexponential fit function [y =A+ C*exp (–t/T2)], where A= Absolute bias, C= signal intensity, T2= transverse relaxation time. Means and standard deviation for T2 relaxation times for each ROI were calculated.
Effect(s) tested	One-way ANOVA (Tukey's post-test) for comparison of mean between two groups was performed. Levene's test was performed for homogeneity of variance analysis.

Specify type of analysis: ☐ Whole brain ☐ ROI-based ☒ Both

Anatomical location(s)	Regions of interest (ROIs) were drawn at desired locations within the brain using an image sequence analysis (ISA) tool package (Paravision 5, Bruker)

Statistic type for inference (See Eklund et al. 2016)	As we use structural, relaxometry and perfusion MRI data this is not relevant.
Correction	As we use structural, relaxometry and perfusion MRI data this is not relevant.

Models & analysis

n/a	Involved in the study
☒	☐ Functional and/or effective connectivity
☒	☐ Graph analysis
☒	☐ Multivariate modeling or predictive analysis
